# Screening of broad-host expression promoters for shuttle expression vectors in non-conventional yeasts and bacteria

**DOI:** 10.1186/s12934-024-02506-x

**Published:** 2024-08-16

**Authors:** Liyun Ji, Shuo Xu, Yue Zhang, Hairong Cheng

**Affiliations:** State Key Laboratory of Microbial Metabolism, and School of Life Sciences and Biotechnology, Shanghai Jiao Tong University, Shanghai, China

**Keywords:** Promoter, Shuttle expression, Non-conventional hosts

## Abstract

**Background:**

Non-conventional yeasts and bacteria gain significance in synthetic biology for their unique metabolic capabilities in converting low-cost renewable feedstocks into valuable products. Improving metabolic pathways and increasing bioproduct yields remain dependent on the strategically use of various promoters in these microbes. The development of broad-spectrum promoter libraries with varying strengths for different hosts is attractive for biosynthetic engineers.

**Results:**

In this study, five *Yarrowia lipolytica* constitutive promoters (*yl.hp4d*, *yl.FBA1in*, *yl.TEF1*, *yl.TDH1*, *yl.EXP1*) and five *Kluyveromyces marxianus* constitutive promoters (*km.PDC1*, *km.FBA1*, *km.TEF1*, *km.TDH3*, *km.ENO1*) were selected to construct promoter-reporter vectors, utilizing α-amylase and red fluorescent protein (RFP) as reporter genes. The promoters' strengths were systematically characterized across *Y*. *lipolytica*, *K. marxianus*, *Pichia pastoris*, *Escherichia coli*, and *Corynebacterium glutamicum*. We discovered that five *K. marxianus* promoters can all express genes in *Y. lipolytica* and that five *Y*. *lipolytica* promoters can all express genes in *K. marxianus* with variable expression strengths. Significantly, the *yl.TEF1* and *km.TEF1* yeast promoters exhibited their adaptability in *P. pastoris*, *E. coli*, and *C. glutamicum*. In yeast *P. pastoris*, the *yl.TEF1* promoter exhibited substantial expression of both amylase and RFP. In bacteria *E. coli* and *C. glutamicum*, the eukaryotic *km.TEF1* promoter demonstrated robust expression of RFP. Significantly, in *E. coli*, The RFP expression strength of the *km*.*TEF1* promoter reached ∼20% of the T7 promoter.

**Conclusion:**

Non-conventional yeast promoters with diverse and cross-domain applicability have great potential for developing innovative and dynamic regulated systems that can effectively manage carbon flux and enhance target bioproduct synthesis across diverse microbial hosts.

**Graphical Abstract:**

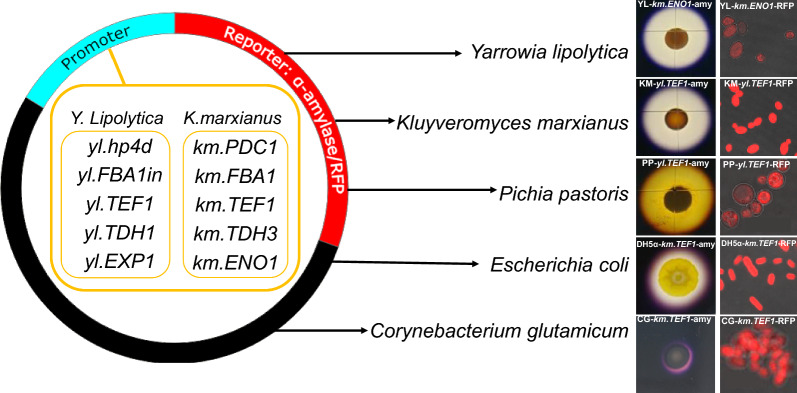

**Supplementary Information:**

The online version contains supplementary material available at 10.1186/s12934-024-02506-x.

## Highlights

- The broad-spectrum promoters enable broad cross-species functionality.
- Five *Kluyveromyces marxianus* promoters (*km.PDC1*, *km.FBA1*, *km.TEF1*, *km.TDH3*, *km.ENO1*) can all express genes in *Yarrowia lipolytica*.
- Five *Y. lipolytica* promoters (*yl.hp4d*, *yl.FBA1in*, *yl.TEF1*, *yl.TDH1*,* yl.EXP1*) can all express genes in *K. marxianus*.
- The *Kluyveromyces marxianus* promoter *km.TEF1* can strongly express RFP in bacteria *E. coli* and *C. glutamicum*.

**Supplementary Information:**

The online version contains supplementary material available at 10.1186/s12934-024-02506-x.

## Introduction

Non-conventional yeasts and bacteria are becoming so prevalent in the fields of biotechnology owing to their unique metabolic capabilities and ability to produce valuable compounds from inexpensive and renewable feedstocks [1, 2]. However, one of the challenges in using these organisms is the absence of appropriate promoters for regulating gene expression in these species. The majority of biotechnology promoters are obtained from model organisms, including *Saccharomyces cerevisiae* and *Escherichia coli *[3, 4].

In the process of bioproduct production, selecting the proper host is a critical phase [5–7]. Promising unconventional chassis cells include the yeasts *Yarrowia lipolytica*, *Kluyveromyces*, and *Pichia pastoris*, and the bacterium *Corynebacterium glutamicum*. *Y*.* lipolytica* is primarily used for the production of proteins, oils, terpenes, organic acids, and sugar alcohols due to the sufficient supply of acetyl-CoA, NADPH and the low glycosylation level of protein [8–10]; *Kluyveromyces marxianus* has shown significant effectiveness in producing aromatic chemicals and biofuel ethanol due to its favorable traits, including the ability to use a wide range of substrates, fast growth, and great resistance to high temperatures [11–14]. *Pichia pastoris* is widely used in the production of heterologous proteins due to high protein secretion capacity and low glycosylation level. *P. pastoris* is also used as a one-carbon carbon source utilization chassis due to the natural methylotrophic characteristics [15]. *Corynebacterium glutamicum* is widely used in the large-scale production of various L-amino acids, such as L-glutamate, L-lysine, L-serine, and L-threonine. *C. glutamicum* is also used to produce organic acids, biofuels, terpenoids and aromatic compounds [16, 17].

A great challenge of bioproducts synthesis is the competition between cell native metabolism pathways and the heterologous target product synthesis pathways for limited cellular carbon resources. The dynamic metabolic engineering is an effective strategy for fine-tuning metabolic flux to maximize target product synthesis [18, 19]. In order to dynamically orchestrate the carbon flux, the heterologous synthesis pathways are often strengthened by engineering promoters and the competitive native pathways are generally altered by knocking out or knocking down [20–24]. However, the competing pathways essential for normal cell growth cannot be completely removed. The dynamic up-regulation and down-regulation on multiple pathways simultaneously could be adjusted by promoter sets with diverse strengths [25–27]. Promoters, the most basic transcriptional regulatory elements, have been used widely for gene expression and metabolic pathway engineering [28–32]. The coordinated co-expression of multiple genes in multistep pathways is required for intricate synthetic biology. Multiple promoters are required for multistep metabolic pathways to avoid repeated usage of the same promoter at adjacent loci. The usage of the same promoter can result in genetic instability of engineered strains due to lost parts of the expression cassettes by loop-out homologous recombination [33, 34]. Replacing promoters with different sequences and regulatory strengths in the functional modules to adjust the adaptability of the chassis cells increases the output of the target products [35–37]. There have been a number of interesting studies on metabolically designed microbial cell factories to generate bio-products with different levels of promoters [38–40]. For example, high-titer production of n-butanol from *E. coli* was achieved by using different expression levels of promoters [38].

Many promoters are incompatible in different hosts [41]. The construction of pre-optimized chassis strains for biosynthesis pathways, promoter substitution, and redesigning, is always required in different hosts. Host specific promoters need to be selected to reconstruct biosynthetic pathways, which is a time-consuming and complicated construction process. Promoters with broad spectrum in different hosts are rare. The development of broad-spectrum promoters could enable synthetic circuit shuttles to be expressed between diverse host cells, from yeast to yeast, or even between the eukaryotic hosts and prokaryotic hosts [42, 43]. The feasibility of some heterologous yeast promoters in different expression systems have been characterized. For example, *Kluyveromyces marxianus TPI* and *Hansenula polymorpha PMA* promoters in *P. pastoris *[44]*,* GAL1/2 promoters from other *Saccharomyces* species in *S. cerevisiae *[34]*, S. cerevisiae* promoters (P_GPD_, P_ADH_, P_TEF_, and P_CYC_) in *K. marxianus *[45] and the eukaryotic promoter GAL1/10 from *S. cerevisiae* direct expressing gene in *E. coli *[46]. The development of promoters with broad host properties could enable rapid phenotyping of genetic constructs in different hosts. Therefore, the strength characterization of different promoters in different hosts is needed for multi-host applications.

In this study, we aimed to find broad-spectrum promoter sets with different strengths to dynamically balance the metabolic flux for the efficient production of high value-added bioproducts in different hosts. We selected five constitutive promoters of *Y*. *lipolytica* and *K. marxianus* respectively to compared promoter strength by the expression levels of reporter genes α-amylase (Oryza sativa, *AMY1A*, 1305 bp) [47] and red fluorescent protein (RFP, *mRuby*, JX489389.1771 bp) [48] in different hosts. The broad-spectrum promoters with different strengths were characterized. Interestingly, we found two yeast promoters that could shuttle express reporter genes in *E. coli, P. pastoris* and *C. glutamicum*. These broad-spectrum promoters will expand the synthetic biology toolbox and the application of bioengineering.

## Materials and methods

### Strains, growth media, and culture conditions

The *Y. lipolytica, K. marxianus, P. pastoris, E. coli* and *C. glutamicum* strains are listed in Table 1. The thermotolerant *Y. lipolytica* CGMCC7326 mutant strain msn4 was used for all the built *Y. lipolytica* transformant strains. The *K. marxianus* CGMCC2.1977 strain was used for all the built *K. marxianus* transformant strains. *P. pastoris* GS115 was used for all the built *P. pastoris* transformant strains. Yeast strains were grown at 30 °C in a YPD medium (10 g/L yeast extract, 5 g/L tryptone, and 20 g/L glucose). When necessary, transformants were screened by adding hygromycin to the YPD. *E. coli* DH5α was used for the amplification of plasmids. *E. coli* BL21 (DE3) was used for plasmid expression. The *E. coli* strains were cultivated at 37 °C in a Luria–Bertani medium (LB) supplemented with ampicillin (100 mg/L) or kanamycin sulfate (50 mg/L). *C. glutamicum* ATCC13032 was grown in LBHIS medium (LB supplemented with brain heart infusion and sorbitol: 5 g/L tryptone, 2.5 g/L yeast extract, 18.5 g/L brain heart infusion broth, 91 g/L sorbitol and 5 g/L NaCl) at 30 °C with chloramphenicol (10 μg/mL) to screen transformants. For solid media, agar (15 g/L) was added.

**Table 1:** Strains used in this study

Strains	Description	Source
Yeast* Y. lipolytica*	msn4 (Thermotolerant	Laboratory storage
	CGMCC7326 mutant)	
Yeast* K. marxianus*	CGMCC2.1977	CGMCC
Yeast* Pichia pastoris*	GS115 (Mut^+^, His^−^)	Invitrogen life
		Technologies
*E. coli* DH5α	*supE44 _lacU169*(_*80lacZ_M15*) *hsdR17*	Thermo fisher scientific
	*recA1 endA1 gyrA96 thi-1 relA1*	
*E. coli* BL21(DE3)	F^*−*^ *ompT hsdS*B*(*r_B_^−^ m_B_^−^*) gal*	Thermo fisher scientific
	*dcm araB::T7RNAP-tetA*	
*Corynebacterium glutamicum*	ATCC13032	ATCC

CGMCC: China General Microbiological Culture Collection Center; ATCC: America Type Culture Collection

### General molecular biology methods

Restriction endonucleases and DNA polymerases were purchased from Thermo Fisher Scientific. High fidelity Taq DNA polymerase (KOD plus, Toyobo) was used for DNA cloning. ExTaq DNA polymerase (Takara) was used for genotype verification. The PCR-amplified products in the agarose gels were purified using a GeneJet Gel Extraction Kit (Thermo Scientific). PCR-amplified products were subcloned into a vector using EasyFusion Assembly Master Mix (New Cell & Molecular Biotech, Suzhou, China). Genewiz (Suzhou, China) performed the primers synthesis.

### Plasmid construction

We selected five constitutive promoters from *Y. lipolytica* and from *K. marxianus* respectively (Table 2) for identification of promoter expression levels in *Y. lipolytica*, *K. marxianus*, *P. pastoris*, *E. coli* and *C. glutamicum*. All plasmids, comprising each promoter and the α-amylase (Oryza sativa, *AMY1A*, 1305 bp), or the RFP gene (*mRuby*, JX489389.1, 771 bp) as reporter genes, were derived from the skeletal plasmid pSWV-*hph* (Fig. S1). The plasmid pSWV-*hph* contains parts of 26S rDNA for integration, hp4d promoter, *aep* terminator, hygromycin resistance gene (*hph*) and ampicillin resistance gene (*amp*^r^) [49] and was obtained from laboratory storage. The plasmid schematic is shown in Fig. S1. The 26S rDNA sequences in shuttle expression vectors are homologous across various yeast strains [50, 51]. The putative promoter regions were amplified by PCR using the primers shown in Table S2 and the genomic *Y. lipolytica* DNA or *K. marxianus* DNA as templates. The promoter sequences and the reporter genes, α-amylase and RFP, are listed in the supplemental material. Detailed information for constructing the plasmids in this study is listed in Table S1. The primers for verifying the constructed plasmids are listed in Table S2.

**Table 2:** List of promoters used in this study

Promoter	Open reading frame regulated	Accession number	bp range	Reference
*Y. lipolytica* constitutive promoters				
* yl.hp4d* (*UAS1B4-leum*)	A hybrid promoter containing four UAS1 tandem elements based on the minimal LEU2 promoter			35
* yl.FBA1in*	Fructose 1,6-bisphosphate aldolase	YALI0E26004g	−826 to + 169	56
* yl.TEF1*	Translation elongation factor EF-1^α^	YALI0C09141p	−418 to −1	30
* yl.TDH1*	Glyceraldehyde-3-phosphate dehydrogenase	YALI0C06369g	−978 to −1	56
* yl.EXP1*	Export protein	YALI0C12034p	−1006 to −1	29
*K. marxianus* constitutive promoters				
* km.PDC1*	Pyruvate decarboxylase	KMXK_0F05000	−998 to −1	57, 58
* km.FBA1*	Fructose 1,6-bisphosphate aldolase	KMXK_0D04110	−932 to −1	57, 58
* km.TEF1*	Translation elongation factor EF alpha-1	KMXK_0G03180	−873 to −1	58
* km.TDH3*	Glyceraldehyde-3-phosphate dehydrogenase isoform 3	KMAR_80062	−534 to −1	57, 58
* km.ENO1*	Enolase	KMXK_0A03750	−726 to −1	57, 58

The elements used in this study are listed with their names, open reading frames regulated, accession numbers, and base pair ranges

The RFP gene and α-amylase gene were inserted into *Nde*I/*Xho*I sites in pET28a to form the plasmids pET28a-rfp and pET28a-amy, respectively. The PCR products for *yl.TEF1*-rfp, *km.TEF1*-rfp, *yl.TEF1*-amy and *km.TEF1*-amy were inserted into *Apa*I/*Hin*dIII sites in pXMJ19 to form plasmids pXMJ19-*yl.TEF1*-rfp, pXMJ19-*km.TEF1*-rfp, pXMJ19-*yl.TEF1*-amy and pXMJ19-*km.TEF1*-amy.

### The non-conventional yeasts and bacterium transformation

Transformant strain details used in this study are shown in Table 3. The PCR products of the constructed promoter-reporter plasmids, with a pair of primers, Broad-host vector-F/ Broad-host vector-R (Table S2), were purified from the agarose gel. Additionally, they were used to transform yeast strains *Y. lipolytica* msn4, *K. marxianus* CGMCC2.1977 and *P. pastoris* GS115. Yeasts were transformed using the lithium acetate method described by Chen et al. [52].

**Table 3:** Transformant strains used in this study

Transformant strains	Description
The *Y. lipolytica* recombinant strains via native promoters (α-amylase gene)	
YL-*yl.hp4d*-amy	26SrDNA-*yl.hp4d*-amy-AEP-*yl.hp4d*-*hph*-cyc1-26SrDNA
YL-*yl.FBA1in*-amy	26SrDNA-*yl.FBA1in*-amy-AEP-*yl.hp4d*-*hph*-cyc1-26SrDNA
YL-*yl.TEF1*-amy	26SrDNA-*yl.TEF1*-amy-AEP-*yl.hp4d*-*hph*-cyc1-26SrDNA
YL-*yl.TDH1*-amy	26SrDNA-*yl.TDH1*-amy-AEP-*yl.hp4d*-*hph*-cyc1-26SrDNA
YL-*yl.EXP1*-amy	26SrDNA-*yl.EXP1*-amy-AEP-*yl.hp4d*-*hph*-cyc1-26SrDNA
The *K. marxianus* recombinant strains via native promoters (α-amylase gene)	
KM-*km.PDC1*-amy	26SrDNA-*km.PDC1*-amy-AEP- *km.FBA1*-*hph*-cyc1-26SrDNA
KM-*km.FBA1*-amy	26SrDNA-*km.FBA1*-amy-AEP- *km.FBA1*-*hph*-cyc1-26SrDNA
KM-*km.TEF1*-amy	26SrDNA-*km.TEF1*-amy-AEP- *km.FBA1*-*hph*-cyc1-26SrDNA
KM-*km.TDH3*-amy	26SrDNA-*km.TDH3*-amy-AEP- *km.FBA1*-*hph*-cyc1-26SrDNA
KM-*km.ENO1*-amy	26SrDNA-*km.ENO1*-amy-AEP- *km.FBA1*-*hph*-cyc1-26SrDNA
The *Y. lipolytica* recombinant strains via *K. marxianus* promoters (α-amylase gene)	
YL-*km.PDC1*-amy	26SrDNA *km.PDC1*-amy-AEP- *km.FBA1*-*hph*-cyc1-26SrDNA
YL-*km.FBA1*-amy	26SrDNA-*km.FBA1*-amy-AEP- *km.FBA1*-*hph*-cyc1-26SrDNA
YL-*km.TEF1*-amy	26SrDNA-*km.TEF1*-amy-AEP- *km.FBA1*-*hph*-cyc1-26SrDNA
YL-*km.TDH3*-amy	26SrDNA-*km.TDH3*-amy-AEP- *km.FBA1*-*hph*-cyc1-26SrDNA
YL-*km.ENO1*-amy	26SrDNA-*km.ENO1*-amy-AEP- *km.FBA1*-*hph*-cyc1-26SrDNA
The *K. marxianus* recombinant strains via *Y. lipolytica* promoters (α-amylase gene)	
KM-*yl.hp4d*-amy	26SrDNA-*yl.hp4d*-amy-AEP-*yl.hp4d*-*hph*-cyc1-26SrDNA
KM-*yl.FBA1in*-amy	26SrDNA-*yl.FBA1in*-amy-AEP-*yl.hp4d*-*hph*-cyc1-26SrDNA
KM-*yl.TEF1*-amy	26SrDNA-*yl.TEF1*-amy-AEP-*yl.hp4d*-*hph*-cyc1-26SrDNA
KM-*yl.TDH1*-amy	26SrDNA-*yl.TDH1*-amy-AEP-*yl.hp4d*-*hph*-cyc1-26SrDNA
KM-*yl.EXP1*-amy	26SrDNA-*yl.EXP1*-amy-AEP-*yl.hp4d*-*hph*-cyc1-26SrDNA
The *Y. lipolytica* recombinant strains via native promoters (RFP gene)	
YL-*yl.hp4d*-rfp	26SrDNA-*yl.hp4d*-rfp-AEP-*yl.hp4d*-*hph*-cyc1-26SrDNA
YL-*yl.FBA1in*-rfp	26SrDNA-*yl.FBA1in*-rfp-AEP-*yl.hp4d*-*hph*-cyc1-26SrDNA
YL-*yl.TEF1*-rfp	26SrDNA-*yl.TEF1*-rfp-AEP-*yl.hp4d*-*hph*-cyc1-26SrDNA
YL-*yl.TDH1*-rfp	26SrDNA-*yl.TDH1*-rfp-AEP-*yl.hp4d*-*hph*-cyc1-26SrDNA
YL-*yl.EXP1*-rfp	26SrDNA-*yl.EXP1*-rfp-AEP-*yl.hp4d*-*hph*-cyc1-26SrDNA
The *K. marxianus* recombinant strains via native promoters (RFP gene)	
KM-*km.PDC1*-rfp	26SrDNA *km.PDC1*-rfp-AEP- *km.FBA1*-*hph*-cyc1-26SrDNA
KM-*km.FBA1*-rfp	26SrDNA-*km.FBA1*-rfp-AEP- *km.FBA1*-*hph*-cyc1-26SrDNA
KM-*km.TEF1*-rfp	26SrDNA-*km.TEF1*-rfp-AEP- *km.FBA1*-*hph*-cyc1-26SrDNA
KM-*km.TDH3*-rfp	26SrDNA-*km.TDH3*-rfp-AEP- *km.FBA1*-*hph*-cyc1-26SrDNA
KM-*km.ENO1*-rfp	26SrDNA-*km.ENO1*-rfp-AEP- *km.FBA1*-*hph*-cyc1-26SrDNA
The *Y. lipolytica* recombinant strains via *K. marxianus* promoters (RFP gene)	
YL-*km.PDC1*-rfp	26SrDNA *km.PDC1*-rfp-AEP- *km.FBA1*-*hph*-cyc1-26SrDNA
YL-*km.FBA1*-rfp	26SrDNA-*km.FBA1*-rfp-AEP- *km.FBA1*-*hph*-cyc1-26SrDNA
YL-*km.TEF1*-rfp	26SrDNA-*km.TEF1*-rfp-AEP- *km.FBA1*-*hph*-cyc1-26SrDNA
YL-*km.TDH3*-rfp	26SrDNA-*km.TDH3*-rfp-AEP- *km.FBA1*-*hph*-cyc1-26SrDNA
YL-*km.ENO1*-rfp	26SrDNA-*km.ENO1*-rfp-AEP- *km.FBA1*-*hph*-cyc1-26SrDNA
The *K. marxianus* recombinant strains via *Y. lipolytica* promoters (RFP gene)	
KM-*yl.hp4d*-rfp	26SrDNA-*yl.hp4d*-rfp-AEP-*yl.hp4d*-*hph*-cyc1-26SrDNA
KM-*yl.FBA1in*-rfp	26SrDNA-*yl.FBA1in*-rfp-AEP-*yl.hp4d*-*hph*-cyc1-26SrDNA
KM-*yl.TEF1*-rfp	26SrDNA-*yl.TEF1*-rfp-AEP-*yl.hp4d*-*hph*-cyc1-26SrDNA
KM-*yl.TDH1*-rfp	26SrDNA-*yl.TDH1*-rfp-AEP-*yl.hp4d*-*hph*-cyc1-26SrDNA
KM-*yl.EXP1*-rfp	26SrDNA-*yl.EXP1*-rfp-AEP-*yl.hp4d*-*hph*-cyc1-26SrDNA
The* E. coli* recombinant strains	
DE3-*km.TEF1*-rfp	26SrDNA-*km.TEF1*-rfp-AEP- *km.FBA1*-*hph*-cyc1-26SrDNA
DH5α-*km.TEF1*-rfp	26SrDNA-*km.TEF1*-rfp-AEP- *km.FBA1*-*hph*-cyc1-26SrDNA
DE3-*yl.TEF1*-amy	26SrDNA-*yl.TEF1*-amy-AEP-*yl.hp4d*-*hph*-cyc1-26SrDNA
DH5α-*yl.TEF1*-amy	26SrDNA-*yl.TEF1*-amy-AEP-*yl.hp4d*-*hph*-cyc1-26SrDNA
DE3-*km.TEF1*-amy	26SrDNA-*km.TEF1*-amy-AEP- *km.FBA1*-*hph*-cyc1-26SrDNA
DH5α-*km.TEF1*-amy	26SrDNA-*km.TEF1*-amy-AEP- *km.FBA1*-*hph*-cyc1-26SrDNA
DE3-pET28a-rfp	T7 promoter-LacI-rfp-T7 terminator-*Kana*^*r*^
DE3-pET28a-amy	T7 promoter-LacI-amy-T7 terminator-*Kana*^*r*^
The *P. pastoris* recombinant strains	
PP-*yl.TEF1*-rfp	26SrDNA-*yl.TEF1*-rfp-AEP-*yl.hp4d*-*hph*-cyc1-26SrDNA
PP-*km.TEF1*-rfp	26SrDNA-*km.TEF1*-rfp-AEP- *km.FBA1*-*hph*-cyc1-26SrDNA
PP-*yl.TEF1*-amy	26SrDNA-*yl.TEF1*-amy-AEP-*yl.hp4d*-*hph*-cyc1-26SrDNA
PP-*km.TEF1*-amy	26SrDNA-*km.TEF1*-amy-AEP- *km.FBA1*-*hph*-cyc1-26SrDNA
The* C. glutamicum* recombinant strains	
CG-pXMJ19-*yl.TEF1*-rfp	*yl.TEF1*-rfp-*Cm*^r^
CG-pXMJ19-*km.TEF1*-rfp	*km.TEF1*-rfp-*Cm*^r^
CG-pXMJ19-*yl.TEF1*-amy	*yl.TEF1*-amy-*Cm*^r^
CG-pXMJ19-*km.TEF1*-amy	*km.TEF1*-amy-*Cm*^r^

YL: *Y. lipolytica*, KM: *K. marxianus*, DE3 and DH5α: *E. coli*, PP: *Pichia pastoris*, CG: *C. glutamicum*; *yl.hp4d*: hybrid promoter contains four UAS1 tandem elements based on the minimal LEU2 promoter (*UAS1B4−leum)*, *yl.FBA1in*: The FBA1in promoter (−826 to +169) containing an intron (+64 to +165) of fructose 1,6−bisphosphate aldolase, *yl.TEF1*: the promoter of translation elongation factor EF−1α, *yl.TDH1*: the promoter of glyceraldehyde−3−phosphate dehydrogenase, *yl.EXP1*: the promoter of export protein, *km.PDC1*: the promoter of pyruvate decarboxylas, *km.FBA1*: the promoter of fructose 1,6−bisphosphate aldolase, *km.TEF1*: the promoter of translation elongation factor EF alpha−1, *km.TDH3*: the promoter of glyceraldehyde−3−phosphate dehydrogenase isoform 3, *km.ENO1*: the promoter of enolase

The yeast strain taken from -70 °C was spread on a YPD plate and incubated at 30 °C for 20 h. The cells were scraped from the surface of the plate and added into a sterile 1.5 mL microcentrifuge tube. In the microcentrifuge tube, cells were in the presence of 82 μL polyethylene glycol 4000 (50%, w/v), 5 μL 2 M dithiothreitol, 3.5 μL 3 M lithium acetate, 5 μL 5.0 mg/mL single-stranded carrier DNA (heated in a boiling water bath for 5 min and then chilled in ice/water) and 5 μL linearized DNA (about 1 μg/μL). The transformation mix was thoroughly vortexed. The tube was incubated at 39 °C for 60 min and then centrifuged at 2000 rpm at room temperature for 5 min. The supernatant was discarded and 500 μL YPD medium was added to suspend the cells. The cells were recovered at 30 °C for 60 min and spread directly on a well-dried selective plate and incubated at 30 °C. The transformant colonies appeared about 48 h after transformation and the colonies were picked and verified with corresponding validation primers (Table S2).

*E. coli* and *C. glutamicum* were transformed using the methods by Hu et al. [53]. Overnight, the *E. coli* culture was inoculated into 50 mL LB media at 37 °C and 200 rpm until OD_600_ reached 0.5. The *E. coli* cells were cooled on ice for 10 min, centrifuged, washed 3 times with ice-cold 0.1 M CaCl_2_, and stored at −70 °C in 1.5 mL aliquots. For transformation, an aliquot of competent cells was thawed on ice and 1–2 μL plasmid was added. The mixture was incubated on ice for 30 min and put in a 42 °C water-bath for 90 s. The mixture was then cooled on ice for 3 min and 400 μL LB media was added. The mixture was incubated at 37 °C and 200 rpm for 1 h and plated on LB agar containing antibiotics for selection.

Overnight, the *C. glutamicum* culture was inoculated into 40 mL of the Epo media (10 g/L tryptone, 5 g/L yeast extract, 10 g/L NaCl, 30 g/L glycine, 1 g/L Tween-80) to an initial OD_600_ of 0.2. The culture was grown at 200 rpm and 30 °C until OD_600_ reached 0.6. The cells were cooled on ice for 15 min, centrifuged, washed 3 times with ice-cold 10% glycerol, and stored at −70 °C in 1.5 mL aliquots. For electro-transformation, an aliquot of the competent cells was thawed on ice and 5 μL plasmid was added. The mixture was transferred to a cold electroporation cuvette (0.1 cm) and electroporated at 1.8 kV with a 5 ms pulse. Immediately after the electroporation, 1 mL LBHIS (5 g/L tryptone, 5 g/L NaCl, 2.5 g/L yeast extract, 18.5 g/L brain heart infusion powder and 91 g/L sorbitol) was added to the cuvette. The mixture was transferred to a 1.5 mL Eppendorf tube, incubated at 30 °C and 200 rpm for 1 h, and plated on LBHIS agar containing antibiotics for selection.

### Methods for amylase activity assays

#### Relative amylase activities with starch-iodine assay

In Fig. 2a, the transformant strains of *Y. lipolytica* and *K. marxianus* with amylase under control of each promoter were spotted on YPD starch agar media (1% soluble starch) and incubated at 30 °C for 3 days or 6 days. The *Y. lipolytica* msn4 and *K. marxianus* strain CGMCC2.1977 were used as the negative controls. Next, the plates were sprayed with an iodine solution. The iodine solution consisted of 25 g iodine into a saturated solution of 10 g potassium in 10 mL distilled water. The solution was stirred and dissolved, then added 500 mL ethanol and added distilled water to 1000 mL. Positive activity was defined as a clear halo around the colony on a purple background. From each transformant strain, 24 colonies were selected, spotted on YPD starch agar, supplemented with hygromycin, and incubated at 30 °C for 3 days or 6 days. Three colonies with the largest clear halos of each transformant strain were selected. The suspensions (100 µL) of serial dilutions (10^–4^ times) of each colony were spread on YPD starch agar media to obtain isolates that stably expressed amylase. Next, the isolates were point inoculated on YPD starch agar media. The transformant strains with relatively strong amylase expression were cultured for 3 days. Strains with weak expression were cultured for 6 days and the three colonies of the transformant strainYL-*km.FBA1*-amy were used as the reference under both culture conditions (Fig. 2a). The diameters of the clear zones over the diameters of the colonies were measured using a ruler. Relative amylase activities of different transformant strains were compared by the Halo: colony ratio [54] (Table 4, Fig. 2b). In Fig. S2, the preliminary starch-iodine assays for different control strains were detected.

**Table 4:** Amylase activities produced by the transformant strains with amylase under control of each promoter

Strains	Halo: Colony ratio^a^	Activity (U/mL)^b^
YL-*yl.hp4d*-amy	3.22 ± 0.15	38.98 ± 2.13
YL-*yl.FBA1in*-amy	2.81 ± 0.17	38.02 ± 0.80
YL-*yl.TEF1*-amy	3.93 ± 0.36	41.69 ± 1.54
YL-*yl.TDH1*-amy	3.83 ± 0.23	41.99 ± 2.54
YL-*yl.EXP1*-amy	4.04 ± 0.28	40.95 ± 1.42
KM-*km.PDC1*-amy	3.38 ± 0.22	40.90 ± 0.83
KM-*km.FBA1*-amy	2.98 ± 0.08	36.67 ± 0.87
KM-*km.TEF1*-amy	3.05 ± 0.04	42.46 ± 1.10
KM-*km.TDH3*-amy	2.48 ± 0.04	38.56 ± 0.78
KM-*km.ENO1*-amy	2.81 ± 0.04	37.73 ± 0.79
YL-*km.PDC1*-amy	2.48 ± 0.59	40.78 ± 1.24
YL-*km.FBA1*-amy	2.21 ± 0.21	37.47 ± 1.12
YL-*km.TEF1*-amy	1.57 ± 0.21	17.29 ± 1.33
YL-*km.TDH3*-amy	2.61 ± 0.10	42.55 ± 0.49
YL-*km.ENO1*-amy	2.89 ± 0.16	44.36 ± 1.03
KM-*yl.hp4d*-amy	1.29 ± 0.14	5.71 ± 0.38
KM-*yl.FBA1in*-amy	0.00 ± 0.00	2.57 ± 0.27
KM-*yl.TEF1*-amy	2.76 ± 0.15	43.65 ± 1.77
KM-*yl.TDH1*-amy	2.71 ± 0.52	46.29 ± 1.53
KM-*yl.EXP1*-amy	1.37 ± 0.06	25.36 ± 2.10

^a^Three colonies for each transformant strain; ^b^Five replicates of the colony with the largest halo:colony ratio for each transformant strain

#### Absolute amylase activities with 3, 5-dinitrosalicylic acid (DNS) reducing sugar assay

The DNS method was used to determine the absolute amylase activity of each transformant strain. For each transformant strain, the three isolates with the highest Halo: colony ratios were chosen and cultured in 5 mL of YPG medium (10 g/L yeast extract, 5 g/L tryptone and 20 g/L glycerol) in a 50 mL tube at 30 °C for three days with rotary shaking at 220 rpm. The clear supernatants (crude extracellular amylase extracts) were obtained after centrifugation at 7800 × g for 10 min at 4 ºC two times.

A reaction mixture of 150 μL crude amylase extract and 300 μL 1% soluble starch solution was incubated in 0.1 M sodium phosphate buffer (pH 7.0) at 45 °C for 60 min. Subsequently, 600 μL DNS solution was added and boiled for 10 min for color development. The absorbance of the mixture was measured at 540 nm and compared to a prepared blank control solution (distilled water instead of crude amylase extract). The glucose concentration of each sample solution and the control solution was obtained from the glucose standard curve. The standard curve was made using 150 μL D-glucose (0.15 mg/mL; 0.3 mg/mL; 0.5 mg/mL; 0.7 mg/mL; 0.9 mg/mL; 1 mg/mL). The glucose content of the sample was subtracted by the glucose content of the control. One unit of the amylase activity was defined as the amount of enzyme required to produce 1 µmol of reducing sugar under the assay conditions described [55] (Fig. 2c). The 540 nm absorbance of five-time diluted DNS reaction mixture of the controls for preliminary amylase activity assays were shown in table S3.

### Quantitative fluorescence measurement and microscopic observation

For quantitative fluorescence measurement, five colonies of each yeast transformant strain with RFP under control of each promoter were cultured in 5 mL of YPD medium in a 50 mL tube at 30 °C for three days. One colony for each *E. coli* transformant strain with RFP under control of each promoter was cultured in 5 mL of LB medium for one day, two days, and three days, at 37 °C. One colony for each *C. glutamicum* transformant strain with RFP under control of each promoter was cultured in 5 mL of LBHIS medium for four days at 30 °C with rotary shaking at 220 rpm. Optical density of cultures, at a wavelength of 600 nm (OD_600_), was measured with an UV-7504 spectrophotometer after dilutions to monitor cell growth. The value of OD_600_ for each colony was measured and diluted to 1. The fluorescence intensity of 1OD for each colony was measured by a multifunctional microplate reader (Spark, TECAN) with monochromator settings as Ex 559 nm/Em 600 nm. The fluorescence intensities of 1OD different control strains for preliminary RFP quantitative fluorescence experiments were showed in table S4. In Fig. 3a, the fluorescence intensity of 1OD for *K. marxianus* strain CGMCC2.1977 was used as the negative control. In Fig. 4c, the fluorescence intensity of 1OD for *E. coli* BL21(DE3) was used as the negative control. In Fig. 6a, the fluorescence intensity of 1OD for *P. pastoris* GS115 and the fluorescence intensity of 1OD for *C. glutamicum* ATCC13032 were used as negative controls for *P. pastoris* transformants and *C. glutamicum* transformants, respectively.

For microscopic observation, among the five colonies of each yeast transformant strain, the one with the highest fluorescence value was selected and cultured in YPD medium. One colony for each *E. coli* transformant strain was cultured in LB medium and one colony for each *C. glutamicum* transformant strain was cultured in LBHIS medium. Confocal images were collected using a confocal microscope (Ti-E Nikon A1R HD25, Tokyo, Japan). In Fig. S3, microscopic RFP fluorescence images of the different control strains were detected.

## Results and discussion

### Strategy for the screening of broad-host expression promoters for construction of broad-host expression vectors

**Fig. 1:**
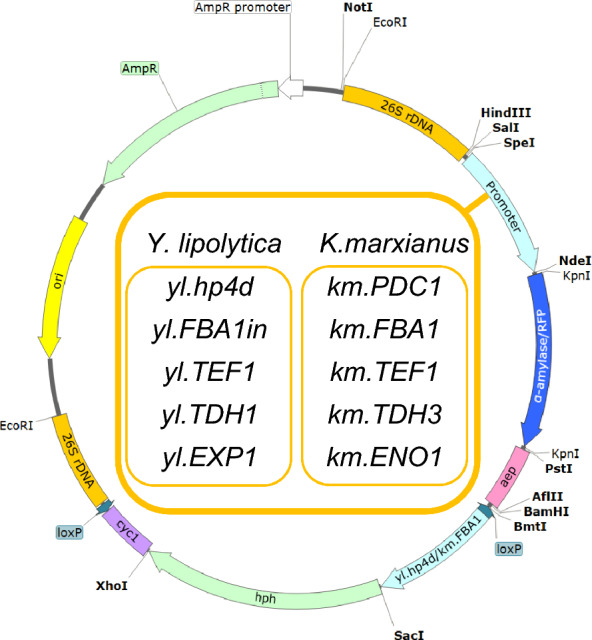
Schematic representation of plasmid construction

Our objective was to evaluate a wide range of promoter sets with varying strengths to create versatile expression vectors and shuttle plasmids to effectively regulate the metabolic flow for the optimal synthesis of valuable bioproducts in various organisms, including non-conventional yeasts (*Y. lipolytica*, *K. marxianus*, *P. pastoris*) and bacteria (*E. coli*, *C. glutamicum*). The strains used in this study listed in Table 1. Five strong constitutive promoters from *Y. lipolytica* and *K. marxianus* respectively were selected to create versatile expression vectors (Table 2). *Y. lipolytica* constitutive promoters included: *yl.hp4d*, a hybrid promoter containing four UAS1 tandem elements based on the minimal LEU2 promoter (*UAS1B4-leum)* [35]; *yl.FBA1in*, the FBA1in promoter (-826 to + 169) containing an intron (+ 64 to + 165) of fructose 1,6-bisphosphate aldolase [56]; *yl.TEF1*, the promoter of translation elongation factor EF-1^α^ [30]; *yl.TDH1*, the promoter of glyceraldehyde-3-phosphate dehydrogenase [56]; *yl.EXP1*, the promoter of export protein [29]. *K. marxianus* constitutive promoters [57, 58] included: *km.PDC1*, the promoter of pyruvate decarboxylase; *km.FBA1*, the promoter of fructose 1,6-bisphosphate aldolase; *km.TEF1*, the promoter of translation elongation factor EF alpha-1; *km.TDH3*, the promoter of glyceraldehyde-3-phosphate dehydrogenase isoform 3; *km.ENO1*, the promoter of enolase. The promoters of *Y. lipolytica* (*yl.hp4d, yl.FBA1in, yl.TEF1, yl.TDH1, yl.EXP1*) and *K. marxianus* (*km.PDC1, km.FBA1, km.TEF1, km.TDH3, km.ENO1*) were used to construct plasmids comprising each promoter and the α-amylase or the RFP gene as reporter genes (Fig. 1). These were subsequently used to transform *Y. lipolytica*, *K. marxianus*, *P. pastoris*, *E. coli* and *C. glutamicum* for analysis of the promoter expression strengths in different transformant strains. Details of the transformant strains used are shown in Table 3. Promoter expression strengths were determined by measuring amylase activity and RFP fluorescence activity of transformant strains.

The transformant strains expressing amylase/RFP in *Y. lipolytica* and *K. marxianus* were classified into four categories (Table 3): (1) the *Y. lipolytica* recombinant strains via its native promoters, including YL-*yl.hp4d*-amy/rfp, YL-*yl.FBA1in*-amy/rfp, YL-*yl.TEF1*-amy/rfp, YL-*yl.TDH1*-amy/rfp and YL-*yl.EXP1*-amy/rfp; (2) the *K. marxianus* recombinant strains via its native promoters, including KM-*km.PDC1*-amy/rfp, KM-*km.FBA1*-amy/rfp, KM-*km.TEF1*-amy/rfp, KM-*km.TDH3*-amy/rfp and KM-*km.ENO1*-amy/rfp; (3) the *Y. lipolytica* recombinant strains via *K. marxianus* promoters, including YL-*km.PDC1*-amy/rfp, YL-*km.FBA1*-amy/rfp, YL-*km.TEF1*-amy/rfp, YL-*km.TDH3*-amy/rfp and YL-*km.ENO1*-amy/rfp; (4) the *K. marxianus* recombinant strains via *Y. lipolytica* promoters, including KM-*yl.hp4d*-amy/rfp, KM-*yl.FBA1in*-amy/rfp, KM-*yl.TEF1*-amy/rfp, KM-*yl.TDH1*-amy/rfp and KM-*yl.EXP1*-amy/rfp.

### Amylase expression under each promoter in *Y. lipolytica and K. marxianus*

We used α-amylase as a reporter gene to examine the expression strengths of the ten promoters in *Y. lipolytica* and *K. marxianus.* The amylase activity is the ability to degrade starch and is easy to measure (see “Materials and methods” section). Thus, amylase is a good candidate for examining the relationship between gene expression and promoter strength. The PCR products of the ten promoter-amylase plasmids were used to transform the non-conventional yeasts *Y. lipolytica* msn4 and *K. marxianus* CGMCC2.1977 to yield twenty transformant strains (Table 3). We used two methods for amylase activity assays: a starch-iodine assay for relative amylase activities and DNS reducing sugar assay for absolute amylase activities.

**Fig. 2:**
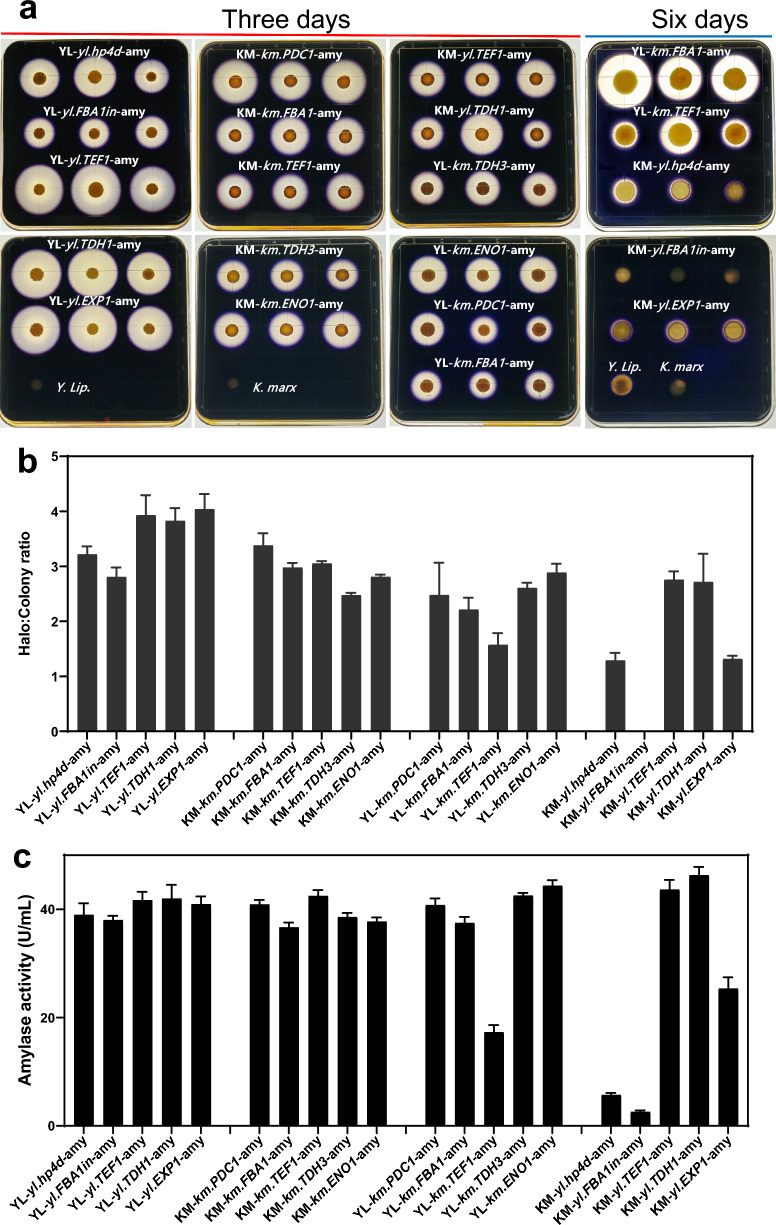
Expression analysis of α-amylase in *Y. lipolytica* and *K. marxianus* transformant strains. **a** Positive amylase activities detected by the clear halos around the colonies of starch-iodine assay, the wild type strains *Y. lipolytica* msn4 and *K. marxianus* CGMCC2.1977 as controls; **b** The mean Halo:Colony ratios (n = 3) of each transformant strain are shown with standard error bars for relative quantifying amylase activities; **c** Absolute amylase activities of the transformant strains were evaluated by DNS reducing sugar assay. Averages of five replicates of each isolate with the highest Halo:Colony ratio for each transformant strain are shown with error bars indicating standard deviation

The starch-iodine assay is useful for rapid screening on the transformants of large populations with high or low amylase activities. Positive amylase active colonies were surrounded by a bright orange halo on YPD starch agar media by spraying iodine solution [54]. Genomic integration mediated by 26S rDNA will cause differences in integration sites and copy numbers, which caused the amylase expression levels for the transformant strain colonies to vary. Despite colony variations, the mean expression levels of the colonies can be used for a rough estimation of expression levels [57]. The three isolates of each transformant strain with the largest clear halos were selected and cultured on YPD starch agar media for 3 or 6 days. The transformant strain, YL-*k*m.*FBA1*-amy, was used as the reference under both culture conditions (3 or 6 days) (Fig. 2a). The relative expression strength of amylase, under control of each promoter in both *Y. lipolytica* and *K. marxianus,* were compared by the Halo: colony ratio (Fig. 2b and Table 4).

We observed that the amylase activities varied with promoter strength in different transformant strains. In the category of the five *Y. lipolytica* recombinant strains expressing amylase via its native promoters, the strains containing *yl.TEF1, yl.TDH1* and *yl.EXP1* had strong expression strengths with the mean Halo:Colony ratios (3.93, 3.83, 4.04 respectively). The strain containing *yl.hp4d* had relatively weaker expression strength with the mean Halo: colony ratio 3.22. The strain containing *yl.FBA1in* had the weakest expression strength with the mean Halo:Colony ratio 2.81. The relative strength is as follows: *yl.TEF1* ∼ *yl.TDH1* ∼ *yl.EXP1* > *yl.hp4d* > *yl.FBA1in*. In the category of the five *K. marxianus* recombinant strains expressing amylase via its native promoters, the strains containing *km.PDC1* and *km.TEF1* had relatively strong expression strengths with the mean Halo: colony ratios (3.38 and 3.05 respectively). The other three strains containing *km.FBA1, km.TDH3* and *km.ENO1* had relatively weaker expression strengths with the mean Halo:Colony ratios (2.98, 2.48, 2.81 respectively). The relative strength is as follows: *km.PDC1* ∼ *km.TEF1* > *km.FBA1*∼ *km.TDH3* ∼ *km.ENO1.*

In the category of the five *Y. lipolytica* recombinant strains via *K. marxianus* promoters, the *Y. lipolytica* strains containing *km.TDH3, km.ENO1, km.PDC1* and *km.FBA1* had strong expression strengths with the mean Halo: colony ratios (2.61, 2.89, 2.48 and 2.21 respectively), which were similar to the ratio of the *Y. lipolytica* strain containing *yl.FBA1in.* The *Y. lipolytica* strain containing *km.TEF1* had very weak expression strength with the mean Halo: colony ratio 1.57. The relative strength is as follows: *km.TDH3* ∼ *km.ENO1* ∼ *km.PDC1*∼ *km.FBA1* >  > *km.TEF1.* In the category of the five *K. marxianus* recombinant strains via *Y. lipolytica* promoters, the *K. marxianus* strains containing *yl.TEF1* and *yl.TDH1* had strong expression strengths with the mean Halo: colony ratios (2.76 and 2.71 respectively), which resembled the *K. marxianus* strains containing *km.FBA1, km.TDH3* and *km.ENO1.* The *K. marxianus* strains containing *yl.hp4d* and *yl.EXP1* showed very low expression with the mean Halo: colony ratios (1.29 and 1.37 respectively). The *K. marxianus* strain containing *yl.FBA1in* in particular couldn’t detect clear halos around the colonies. The relative strength is as follows: *yl.TEF1* ∼ *yl.TDH1* >  > *yl.hp4d* ∼ *yl.EXP1* > *yl.FBA1in.*

Five replicates of each isolate with the highest Halo:Colony ratio for each transformant strain were cultured in YPG medium for three days with rotary shaking for absolute amylase activity quantification in liquid cultures using the DNS method (see "Materials and methods" section) [55]. The results of the DNS reducing sugar assay (Table 4 and Fig. 2c) aligned with the Halo:Colony ratio results of starch-iodine assay (Table 4 and Fig. 2b) with only slight differences. This may be the results of the starch-iodine assay were the average value of amylase activities expressed by three different colonies of each transformant strain. In Fig. 2c, the *Y. lipolytica* strains expressing amylase via its native promoters showed high amylase activities ranging from 38.02 U/mL to 41.99 U/mL. The *K. marxianus* strains expressing amylase via its native promoters also showed high amylase activities ranging from 36.67 U/mL to 42.46 U/mL. The *Y. lipolytica* strains containing *km.PDC1, km.FBA1, km.TDH3, km.ENO1* and the *K. marxianus* strains containing *yl.TEF1, yl.TDH1* had high amylase activities (37.73 U/mL, 40.78 U/mL, 42.55 U/mL, 44.36 U/mL, 43.65 U/mL, 46.29 U/mL respectively), which resembled the *Y. lipolytica* strains and the *K. marxianus* strains expressing amylase via their native promoters. The *Y. lipolytica* strains containing *km.TEF1* and the *K. marxianus* strains containing *yl.hp4d, yl.FBA1in, yl.EXP1* showed very weak amylase expression with very low amylase activities at 17.29 U/mL, 5.71 U/mL, 2.57 U/mL and 19.6 U/mL respectively.

The results showed that the five *K. marxianus* promoters in *Y. lipolytica* and the five *Y. lipolytica* promoters in *K. marxianus* can all express α-amylase with variable expression strength. The promoters *km.PDC1, km.FBA1, km.TDH3, km.ENO1, yl.TEF1, yl.TDH1*, highly express amylase in both *Y. lipolytica* and *K. marxianus*, can be used as the broad-spectrum promoters for construction of broad-host expression vectors to express heterologous synthesis pathways in different hosts and to assess appropriate expression chassis. The weak amylase expression promoters, *km.TEF1* in *Y. lipolytica*, *yl.hp4d*, *yl.FBA1in* and *yl.EXP1* in *K. marxianus,* can be used to express the metabolic flow essential for host growth and competitive for the heterologous metabolic pathway.

### RFP expression under each promoter in *Y. lipolytica and K. marxianus*

We used RFP gene as the reporter gene to examine how the RFP expression varied with the strengths of the ten promoters in *Y. lipolytica* and *K. marxianus*. The RFP fluorescence is easy to detect and quantify in different hosts. We used two methods to check RFP gene expression. The fluorescence intensity for each transformant strain was measured by a multifunctional microplate reader to quantify RFP gene expression levels. Confocal images were collected using a confocal microscope for visual and qualitative view of RFP gene expression.

**Fig. 3:**
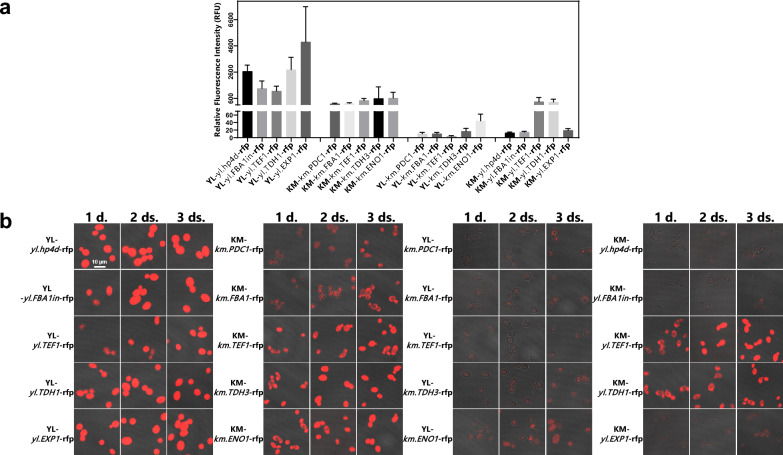
Expression analysis of RFP in *Y. lipolytica* and *K. marxianus* transformant strains. **a** Mean RFP fluorescence intensities (RFU) of the colonies (n = 5) of each *Y. lipolytica* or *K. marxianus* transformant strain with reporter gene RFP under control of each promoter cultured for three days are shown with standard errors (gain value 80); **b** Microscopic fluorescence images of the colony with the highest fluorescence value of each *Y. lipolytica* or *K. marxianus* transformant strain cultured for one day, two days, three days. Fluorescent images of the strains were taken in the same setting

For quantitative fluorescence measurement, the 1OD fluorescence intensities (RFU) for five colonies of each transformant strain were measured by a multifunctional microplate reader with PMT (photomultiplier tube) gain value 80 (Table 5 and Fig. 3a). The fluorescence intensity of 1OD for *K. marxianus* strain CGMCC2.1977 was used as the negative control. The relative fluorescence intensities of the samples were subtracted by the fluorescence intensity of the control. The five *Y. lipolytica* recombinant strains expressing RFP via its native promoters showed drastically high fluorescence intensities compared to the other three categories. The strain containing *yl.EXP1* had the highest RFP expression with the mean fluorescence intensity 4901.2 RFU. The strains containing *yl.hp4d* and *yl.TDH1* had relatively weaker RFP expression with the mean fluorescence intensities of 2671.1 RFU and 2795.2 RFU, respectively. The strains containing *yl.FBA1in* and *yl.TEF1* had the weakest RFP expressions with the mean fluorescence intensities 1358.5 RFU and 1162.8 RFU, respectively. The relative strength is as follows: *yl.EXP1* >  > *yl.TDH1* ∼*yl.hp4d* >  > *yl.FBA1in* ∼ *yl.TEF1*. In the category of the *K. marxianus* recombinant strains expressing RFP via its native promoters, the strains containing *km.TEF1, km.ENO1* and *km.TDH3* had relatively strong RFP expression with the mean fluorescence intensities 444.7 RFU, 598.2 RFU, and 627.6 RFU, respectively. The strains containing *km.PDC1* and *km.FBA1* had relatively weak RFP expression with mean fluorescence intensities of 191.4 RFU and 221.0 RFU, respectively. The relative strength is as follows: *km.ENO1* ∼ *km.TDH3* > *km.TEF1* > *km.FBA1* ∼ *km.PDC1*. The five *Y. lipolytica* recombinant strains expressing RFP via *K. marxianus* promoters had extremely lower fluorescence intensities compared to the other three categories ranging from 3.8 RFU to 44.6 RFU. In the category of the five *K. marxianus* recombinant strains expressing RFP via *Y. lipolytica* promoters, the *K. marxianus* strains containing *yl.TEF1* and *yl.TDH1* showed strong fluorescence intensities at 351.5 RFU and 326.6 RFU, respectively. This was comparable to the fluorescence intensity of the *K. marxianus* strain containing *km.TEF1* at 444.7 RFU. The *K. marxianus* strains containing *yl.hp4d*, *yl.TDH1* and *yl.EXP1* had weak expression strength with mean fluorescence intensities of 12.9 RFU, 14.8 RFU and 19.6 RFU, respectively. The relative strength is as follows: *yl.TEF1* ∼ *yl.TDH1* >  > *yl.hp4d* ∼ *yl.TDH1* ∼ *yl.EXP1.*

**Table 5:** RFU of the transformant strains with reporter gene RFP under control of each promoter (Gain value 80)

Strains	Fluorescence intensities (RFU)
YL-*yl.hp4d*-rfp	2671.1 ± 459.9
YL-*yl.FBA1in*-rfp	1358.5 ± 561.5
YL-*yl.TEF1*-rfp	1162.8 ± 368.4
YL-*yl.TDH1*-rfp	*2795.2* ± 934.0
YL-*yl.EXP1*-rfp	*4901.2* ± 2688.6
KM-*km.PDC1*-rfp	191.4 ± 48.4
KM-*km.FBA1*-rfp	221.0 ± 44.9
KM-*km.TEF1*-rfp	444.7 ± 147.9
KM-*km.TDH3*-rfp	598.2 ± 878.9
KM-*km.ENO1*-rfp	627.6 ± 436.2
YL-*km.PDC1*-rfp	10.6 ± 3.7
YL-*km.FBA1*-rfp	10.4 ± 3.3
YL-*km.TEF1*-rfp	3.8 ± 1.7
YL-*km.TDH3*-rfp	17.0 ± 7.8
YL-*km.ENO1*-rfp	44.6 ± 18.0
KM-*yl.hp4d*-rfp	12.9 ± 2.2
KM-*yl.FBA1in*-rfp	14.8 ± 2.1
KM-*yl.TEF1*-rfp	351.5 ± 318.7
KM-*yl.TDH1*-rfp	326.6 ± 213.0
KM-*yl.EXP1*-rfp	19.6 ± 4.3

Among the five colonies of each transformant strain, the one with the highest fluorescence value was selected and cultured in YPD medium for one day, two days and three days for confocal microscopy. The confocal images for these transformant strains are shown in Fig. 3b. These transformant strains showed red fluorescence in cytosol and the red fluorescence brightness was different among the promoters used. The red fluorescence brightness of the confocal image for each transformant strain increased from the first day to the third day. In the category of the five *Y. lipolytica* recombinant strains expressing RFP via its native promoters, the red fluorescence brightness of the strains containing *yl.hp4d*, *yl.TDH1* and *yl.EXP1* were very high on the first day and the red fluorescence brightness of the strains containing *yl.FBA1in* and *yl.TEF1* were relatively weak. In the category of the five *K. marxianus* recombinant strains expressing RFP via its native promoters, the red fluorescence brightness of the strains containing *km.TEF1, km.TDH3* and *km.ENO1* were highest. The red fluorescence of the strains containing *km.PDC1* and *km.FBA1* were never bright. The red fluorescence of the category of the five *Y. lipolytica* recombinant strains expressing RFP via *K. marxianus* promoters had the lowest brightness compared to the other three categories. In the category of the five *K. marxianus* recombinant strains expressing RFP via *Y. lipolytica* promoters, the high red fluorescence brightness of the *K. marxianus* strains containing *yl.TEF1* and *yl.TDH1* were comparable to the *K. marxianus* strains containing *km.TEF1*, *km.TDH3* and *km.ENO1*. The *K. marxianus* strains containing *yl.hp4d*, *yl.TDH1* and *yl.EXP1* had the weakest brightness.

The results showed that the five *K. marxianus* promoters all can express RFP in *Y. lipolytica* and the five *Y. lipolytica* promoters also all can express RFP in *K. marxianus* with variable expression strength. In our study, the five *K. marxianus* promoters *km.PDC1, km.FBA1, km.TEF1, km.TDH3* and *km.ENO1* did not highly express RFP in *Y. lipolytica* and did not coordinate with α-amylase expression. The *Y. lipolytica* promoters *yl.TEF1* and *yl.TDH1* have the potential to highly express amylase and RFP in *K. marxianus.* The *K. marxianus* promoters *km.PDC1, km.FBA1, km.TDH3* and *km.ENO1* only have the potential to highly express amylase in *Y. lipolytica.* The *Y. lipolytica* promoters *yl.hp4d*, *yl.FBA1in,* and *yl.EXP1* could weakly express RFP in *K. marxianus*, which coordinates with α-amylase expression. Our results revealed that the correlation between α-amylase expression and RFP expression in each transformant strain was weak. These results underscore that gene expression is not always linearly related to promoter strength, which may vary and depend on the specific gene.

**Fig. 4:**
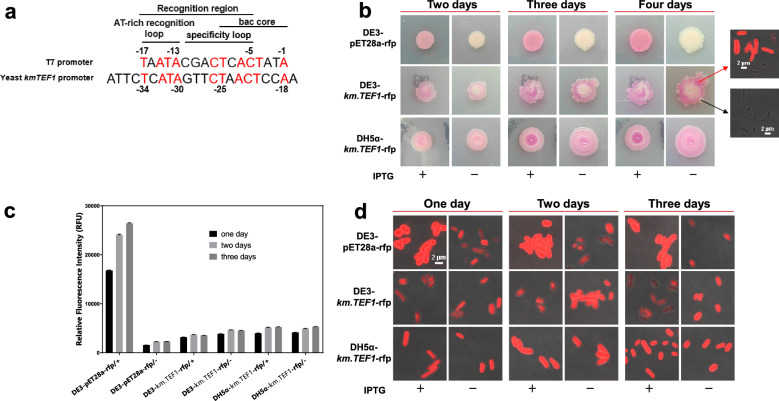
Yeast shuttle vectors express RFP in *Escherichia coli*. **a** Comparison of the yeast *km.TEF1* promoter and the bacteriophage T7 RNAP promoter. All sequences shown in the 5’-3’ orientation. The yeast *km.TEF1* promoter sequence is aligned with the bacteriophage T7 RNAP promoter sequence to highlight analogous positions relative to transcription initiation. **b** Each transformant strain spotted on LB agar medium with or without 100 μM IPTG for two days, three days and four days. The obvious red color of the colonies was observed; **c** RFP fluorescence of each transformant strain cultured for one day, two days, three days was measured in three wells in a 96-well plate. The means (three replicates) and the standard deviations were shown (gain value 70); **d** Microscopic fluorescence images of each transformant strain cultured for one day, two days, three days. “ + ” means with IPTG, “-” means without IPTG

**Fig. 5:**
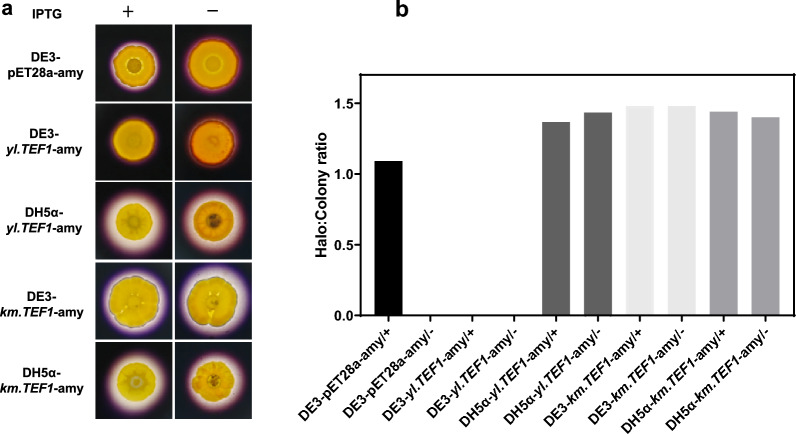
Yeast shuttle vectors express α-amylase in *Escherichia coli*. **a** Positive amylase activities detected by the clear halos around the colonies of starch-iodine assay; **b** Halo diameter to colony diameter ratios of the transformant strains for relative quantifying amylase activities. “ + ” means with IPTG, “-” means without IPTG

In most cases, gene expression and activity were correlated with promoter strength [29, 35, 59]. However, the stronger promoters are not always better for expressing different exogenous genes. For example, the strong T7 native promoter was also not always better for expressing different exogenous genes in *E. coli*. In our results (Sect. "Yeast shuttle vectors expressed in *Escherichia coli*"), T7 promoter could strongly express RFP (Fig. 4), but could only weakly express amylase (Fig. 5) in *E. coli*. The different expression levels of RFP and α-amylase under the control of T7 promoter in *E. coli* further verified that the gene expression level by the same promoter depends on the specific gene.

### Yeast shuttle vectors expressed in *Escherichiacoli*

We discovered that the *km.TEF1* promoter from *K. marxianus* could be used for shuttle expression in *E. coli*. During the cloning of plasmid p*km.TEF1*-rfp (Table S1) in *E. coli* DH5α, we observed that the colonies of the *E. coli* DH5α transformant strain containing plasmid p*km.TEF1*-rfp would turn red in color. The finding suggests that the eukaryotic *km*.*TEF1* promoter from *K. marxianus* has the ability to allow gene expression in the prokaryotic host *E. coli*. A similar study reports that the eukaryotic promoter GAL1/10 from *S. cerevisiae* could directly express genes in the *E. coli *[46]*.* Any piece of random DNA unlikely to be a functional promoter is not that far from a functional bacterial promoter. A single mutation for each of the evolved random sequences was found to confer the promoter function and can be further increased in a stepwise manner by additional mutations that improve similarity to canonical promoters in *E. coli* [60]. So, the *km.TEF1* promoter from *K. marxianus* may happen to have similar elements to bacterial promoters. In Fig. 4a, we used the well characterized bacteriophage T7 promoter sequence as a reference for comparison with the eukaryotic *km*.*TEF1* promoter sequence from *K. marxianus.* The −34 to −18 positions of the yeast *km*.*TEF1* promoter sequence has similarity with the T7 promoter, suggesting a common promoter function of this region as T7 promoter. The recognition region (positions −17 to −5) of the T7 native promoter includes the AT-rich recognition loop (positions −17 to −13) and the specificity loop (positions −12 to −5). These provide a sequence-specific recognition by the bacteriophage T7 RNA-polymerase (RNAP) [61]. The T7 RNAP can recognize the sequences closely related to the T7 native promoter [62]. The *km*.*TEF1* promoter shares the similar sequences of the AT-rich recognition loop (positions −34 to −30), the specificity loop (positions −29 to −22) and the bacteriophage core region (positions −25 to −18) with T7 promoter.

To further characterize the behavior of the *km.TEF1* promoter in *E. coli*, we also transformed *E. coli* BL21 (DE3) with plasmid p*km.TEF1*-rfp to yield the transformant strain DE3-*km.TEF1*-rfp, and we transformed *E. coli* DE3 with plasmid pET28a-rfp to yield the transformant strain DE3-pET28a-rfp for comparing to the most studied T7 expression system. The skeletal plasmid pSWV-*hph *[63] used for constructing the plasmid p*km.TEF1*-gene and the pET28a used for constructing the plasmid pET28a-gene with the same origin PBR322 of *E. coli* are high-copy-number plasmid [64]. In Fig. 4b, the transformant strains DE3-pET28a-rfp, DE3-*km.TEF1*-rfp and DH5α-*km.TEF1*-rfp were spotted on the LB agar medium with or without 100 μM IPTG for two days, three days, and four days. Obvious red color of the strain DE3-pET28a-rfp was observed when induced by IPTG, while the strain DE3-pET28a-rfp without IPTG was white and vaguely red. The strains DE3-*km.TEF1*-rfp and DH5α-*km.TEF1*-rfp with or without IPTG all showed obvious red color and red color darkened with the increase of days. Only the strain DE3-*km.TEF1*-rfp would differentiate into some white color colonies. Confocal images of the white colonies and red colonies showed that there was no red fluorescence in the white colonies. Additionally, some of the red colonies no longer expressed RFP. This may be because the native plasmid of *E. coli* DE3 cannot coexist with the plasmid p*km.TEF1*-rfp, leading to the loss of exogenous plasmids with the prolongation of growth time.

The transformant strains DE3-pET28a-rfp, DE3-*km.TEF1*-rfp, and DH5α-*km.TEF1*-rfp were also cultured in liquid LB medium with or without 100 μM IPTG for one day, two days and three days to measure fluorescence intensity (Fig. 4c) and confocal microscopy (Fig. 4d). In Fig. 4c, the fluorescence intensity of 1 OD_600_ each transformant strain was measured with PMT gain value 70. The T7 promoter was so strong that RFP fluorescence exceeded the measurable range. Therefore, we lowered the gain value from 80 to 70. The fluorescence intensity of each transformant strain for one day, two days, and three days became higher. The difference between the second day and the third day was not evident. DE3-pET28a-rfp with RFP under control of the T7 promoter had the strongest fluorescence intensity (more than 20,000 RFU on the second day) under the induction of IPTG. RFP expression of the strain DE3-pET28a-rfp without IPTG induction was the weakest (about 2000 RFU on the second day). The promoter *km*.*TEF1* can express RFP in both *E. coli* DE3 and *E. coli* DH5α. The fluorescence intensities of the strains DE3-*km.TEF1*-rfp and DH5α-*km.TEF1*-rfp with or without IPTG were about 4000 or 5000 RFU with no significant difference among them. This indicates that the promoter, *km*.*TEF1,* should be classified as a strong constitutive promoter in *E. coli*. In Fig. 4d, the confocal images showed that these transformant strains had strong RFP expression and the red fluorescence was already very high on the first day.

We transformed the plasmids, p*yl.TEF1*-amy and p*km.TEF1*-amy, with amylase under control of the promoters *yl*.*TEF1* and *km*.*TEF1,* respectively, into *E. coli* DE3 and DH5α to further confirm whether the yeast promoters could be used to drive gene expression in *E. coli*. We transformed *E. coli* DE3 with plasmid pET28a-amy to be able to compare the T7 expression system. In Fig. 5a, the transformant strains DE3-pET28a-amy, DE3-p*yl.TEF1*-amy, DH5α-p*yl.TEF1*-amy, DE3-*km.TEF1*-amy, and DH5α-*km.TEF1*-amy were spotted on LB starch agar medium with or without 100 μM IPTG for four days. Then the plates were sprayed with iodine solution. The strain DE3-pET28a-amy with amylase under control of the T7 promoter had a small clear halo around the colony under the induction of IPTG and had no clear halo without IPTG induction. This indicates that the T7 promoter could not express amylase well in *E. coli*. The strain DE3-*yl.TEF1*-amy with or without IPTG had no clear halo, indicating that the promoter *yl*.*TEF1* failed to express amylase in *E. coli* DE3. The strain DH5α-*yl.TEF1*-amy with or without IPTG had clear halos around the colonies, indicating that the promoter *yl*.*TEF1* could express amylase in *E. coli* DH5α. The strains DE3-*km.TEF1*-amy and DH5α-*km.TEF1*-amy with or without IPTG all had clear halos around the colonies, indicating that the promoter *km*.*TEF1* could express amylase in both DE3 and DH5α. The clear halos around the colonies of the strains DH5α-*yl.TEF1*-amy, DE3-*km.TEF1*-amy and DH5α-*km.TEF1*-amy with or without IPTG were approximately the same size and larger than the strain DE3-pET28a-amy with IPTG. The Halo:Colony ratio for each transformant strain was measured to quantify amylase activity (Fig. 5b).

The results of the RFP expression strength showed that the promoter *km*.*TEF1* could reach ~ 20% of the T7 promoter in *E. coli*. The expression of RFP and α-amylase by the promoter *km*.*TEF1* in *E. coli* was not affected by the inducer IPTG. This indicated that the promoter *km*.*TEF1* is a constitutive promoter in *E. coli*. The expression of α-amylase by the promoter *yl*.*TEF1* in *E. coli* was also not affected by the inducer IPTG, indicating that the promoter *yl*.*TEF1* is also a constitutive promoter in *E. coli*. The α-amylase expression was not high in *E. coli* under the control of T7 promoter, *yl*.*TEF1* promoter and *km*.*TEF1* promoter. The different expression levels of RFP and α-amylase under the control of T7 promoter or *km*.*TEF1* promoter in *E. coli* further verified that the gene expression level by the same promoter depends on the specific gene.

### Yeast shuttle vectors expressed in *P. pastoris *and* C. glutamicum*

The *TEF1* promoter from *Ashbya gossypii* functions well in several other yeasts, including *K. marxianus* [65]*.* So, the two promoters *yl*.*TEF1* and *km*.*TEF1* may have the same well functions in other hosts. To further characterize the behaviors of the two promoters *yl*.*TEF1* and *km*.*TEF1* in other yeast and bacterium, we selected the non-conventional yeast *P. pastoris* GS115 and bacterium *C. glutamicum* ATCC13032 for transformation. The PCR products of the plasmids p*yl*.*TEF1-*rfp, p*km*.*TEF1-*rfp, p*yl*.*TEF1-*amy, p*km*.*TEF1-*amy (Table S1) were transformed into *P. pastoris* GS115 to yield the *P. pastoris* transformant strains PP-*yl.TEF1*-rfp, PP-*km.TEF1*-rfp, PP-*yl*.*TEF1-*amy and PP-*km*.*TEF1-*amy (Table 2). The plasmids pXMJ19-*yl.TEF1*-rfp, pXMJ19-*km.TEF1*-rfp, pXMJ19-*yl.TEF1*-amy and pXMJ19-*km.TEF1*-amy (Table S1) were transformed into *C. glutamicum* ATCC13032 to yield the *C. glutamicum* transformant strains CG-*yl.TEF1*-rfp, CG-*km.TEF1*-rfp, CG-*yl*.*TEF1-*amy and CG-*km*.*TEF1-*amy (Table 3). The pXMJ19 used for constructing the plasmids pXMJ19-*yl.TEF1*-gene and pXMJ19-*km.TEF1*-gene with the origin pBL1 of *C. glutamicum* is the high-copy-number plasmid [66].

**Fig. 6:**
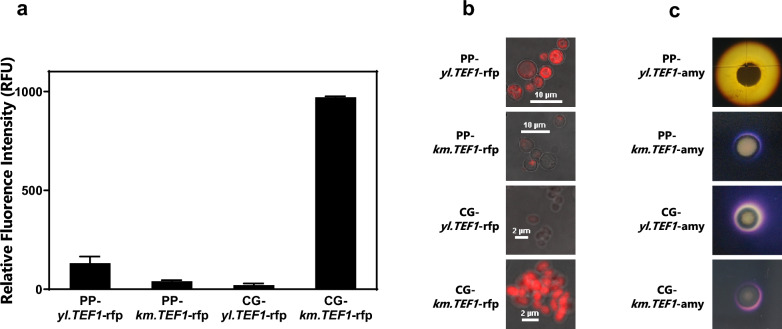
Yeast Shuttle Vectors Express RFP and α-amylase in *P. pastoris* and *C. glutamicum*. **a** RFP fluorescence of each transformant strain cultured for three days was measured in three wells in a 96-well plate. Five colonies were selected for each *P. pastoris* transformant strain and one colony was selected for each *C. glutamicum* transformant strain. The means and standard deviations were shown (gain value 80); **b** Microscopic fluorescence images of each transformant strain cultured for three days; **c** Positive amylase activities detected by the clear halos around the colonies of starch-iodine assay

We selected five colonies from each of the two *P. pastoris* transformant strains, PP-*yl.TEF1*-rfp and PP-*km.TEF1*-rfp, cultured in YPD medium for three days with rotary shaking. One colony from each of the two *C. glutamicum* transformant strains CG-*yl.TEF1*-rfp and CG-*km.TEF1*-rfp was selected and cultured in LBHIS medium for four days with rotary shaking. In Fig. 6a, the 1 OD_600_ fluorescence intensity of each transformant strain was measured with gain value 80. The strain PP-*yl.TEF1*-rfp containing *yl.TEF1* and the strain PP-*km.TEF1*-rfp containing *km.TEF1* could express RFP in *P. pastoris* with the mean fluorescence intensities 131.1 and 40.3 RFU, respectively*.* The strain CG-*km.TEF1*-rfp containing *km.TEF1* had the highest RFP expression level (970.3 RFU). The strain CG-*yl.TEF1*-rfp had the weakest RFP expression level (20.7 RFU). In Fig. 6b, the one had the highest fluorescence value for each of the two strains PP-*yl.TEF1*-rfp and PP-*km.TEF1*-rfp was selected for confocal microscopy. The strains PP-*yl.TEF1*-rfp and CG-*km.TEF1*-rfp had high red fluorescence brightness, while the strains PP-*km.TEF1*-rfp and CG-*yl.TEF1*-rfp had weak red fluorescence brightness.

The results showed that the *Y. lipolytica* promoter *yl.TEF1* highly expressed α-amylase and RFP in yeast *K. marxianus* and in yeast *P. pastoris.* The *K. marxianus* promoter *km.TEF1* highly expressed RFP in bacterium *E. coli* and in bacterium *C. glutamicum.* The red fluorescence in the bacterium *C. glutamicum* strain CG-*km.TEF1*-rfp was more than 5 times lower than the *E. coli* strains DE3-*km.TEF1*-rfp and DH5α-*km.TEF1*-rfp. The plasmid p*km.TEF1*-rfp exhibited a higher level of red fluorescent protein expression in *E. coli* compared to the plasmid pXMJ19-*km.TEF1*-rfp in *C. glutamicum*. This suggests that although the *km.TEF1* promoter was capable of expressing red fluorescent protein in both bacteria, its expression was more robust in *E. coli.* It is not surprising that these promoters function across yeasts and bacteria, since yeast promoters tend to be transferable across yeasts within a certain genetic distance [67, 68] and that bacterial promoters could possibly exist by chance within yeast promoters. It is still useful that these particular sequences were found to function across hosts. Long nucleosome free regions (NFR) in promoters were evolutionarily conserved. The conserved NFR sequences included the transcription factor binding sites and multiple stretches of poly-A or poly-T. This may be one explanation for some promoters functioning across hosts [69].

## Conclusion

The development of broad-spectrum promoter libraries comprising promoters of varying strengths for different hosts are attractive and meaningful to biosynthetic engineers. As there is no pattern to what promoters will be active in another host. There is also unpredictability when using different genes of interest. So, for gene expression, a large number of different promoters need to be screened. In this study, we found that the five *K. marxianus* promoters in *Y. lipolytica* and the five *Y. lipolytica* promoters in *K. marxianus* could all express α-amylase and RFP with variable expression strength. In addition, the *yl.TEF1* and *km.TEF1* yeast promoters exhibited their adaptability by promoting gene expression in *P. pastoris*, *E. coli*, and *C. glutamicum*. It is worth mentioning that the yeast *P. pastoris* displayed strong expression of amylase and RFP in response to the *yl.TEF1* promoter. On the other hand, both *E. coli* and *C. glutamicum* bacteria exhibited robust synthesis of RFP in response to the eukaryotic *km.TEF1* promoter. It is interesting that the RFP gene expression level of the *km.TEF1* promoter reached 20% of the T7 promoter in *E. coli.* These results suggest that actively controlled strategies to optimize carbon flow and enhance bioproduct synthesis in numerous microbial species are possibly feasible by the distinctive capabilities of non-conventional yeast promoters. Notwithstanding these pioneering discoveries, the research acknowledges specific constraints. Only two visible reporter genes (α-amylase and RFP) were tested. The gene expression level was not always correlated with promoter strength and depends on the specific gene. The reliabilities of these promoters across hosts need to be further verified with additional reporter genes. Through the novel implementation of broad-spectrum promoters, this study has the capacity to significantly advance the development of adaptable, dynamically controlled systems in different hosts. These promoters, having the broad-host range expression potentials, might improve bioproduction efficiency and versatility by optimally controlling pathways of engineering.

### Supplementary Information

Supplementary Material 1.

## Data Availability

No datasets were generated or analysed during the current study.
