# Supplementary material for: Screening of broad-host expression promoters for shuttle expression vectors in non-conventional yeasts and bacteria
Source: Microb Cell Fact. 2024 Aug 16;23:230. doi: 10.1186/s12934-024-02506-x (PMC11330142; doi:10.1186/s12934-024-02506-x)
Supplement: Supplementary file 1 — Supplementary Material 1. [file 12934_2024_2506_MOESM1_ESM.docx]

Supporting information for

**Screening of broad-host expression promoters for shuttle expression vectors in non-conventional yeasts and bacteria**

Liyun Ji^a^, Shuo Xu^a^, Yue Zhang^a^, Hairong Cheng^a*^

^a^ State Key Laboratory of Microbial Metabolism, and School of Life Sciences and Biotechnology, Shanghai Jiao Tong University, Shanghai, China.

**Authors email:**

Liyun Ji jly0091@163.com

Shuo Xu: xushuo@sjtu.edu.cn

Yue Zhang: yyuezhang@sjtu.edu.cn

Hairong Cheng: chrqrq@hotmail.com

^*^Corresponding author email: chrqrq@hotmail.com

**Table and Figure legends**

**Table S1** Plasmids used in this study.

**Table S2** Primers used in this study.

**Table S3** The 540 nm absorbance of five-time diluted DNS reaction mixture of the controls for preliminary amylase activity assays.

**Table S4** The fluorescence intensity of 1OD different control strains for preliminary RFP quantitative fluorescence experiments.

**Fig. S1** Schematic representation of the plasmid pSWV-*hph.*

**Fig. S2** The preliminary starch-iodine assay for different control strains. Positive amylase activities detected by the clear halos around the colonies.

**Fig. S3** Microscopic RFP fluorescence images of the different control strains. Fluorescent images of the strains were taken in the same setting.

**Table S1** Plasmids used in this study.

| **plasmids** | **Description** | **Source** |
| --- | --- | --- |
| pSWV-*hph* | hp4d promoter*, Amp*^r^*, hph* | laboratory storage/ |
| pET28a | LacI, T7 promoter, *Kana^r^* | Novagen |
| pXMJ19 | *Cm^r^* | laboratory storage |
| p*yl.FBA1in* | *yl.FBA1in* promoter inserted into *Spe*I/*Nde*I in pSWV-*hph* | This study |
| p*km.PDC1* | *km.PDC1* promoter inserted into *Spe*I/*Nde*I in pSWV-*hph* | This study |
| p*km.PDC1-km.FBA1* | *km.FBA1* promoter inserted into *Sna*BI/*Sac*I in p*km.PDC1* | This study |
| p*yl.hp4d*-amy | Amylase gene inserted into *Kpn*I in pSWV-*hph* | This study |
| p*yl.FBA1in*-amy | *yl.FBA1in* promoter inserted into *Spe*I/*Nde*I in p*yl.hp4d*-amy | This study |
| p*yl.TEF1*-amy | *yl.TEF1* promoter inserted into *Spe*I/*Nde*I in p*yl.hp4d*-amy | This study |
| p*yl.TDH1*-amy | *yl.TDH1* promoter inserted into *Spe*I/*Nde*I in p*yl.hp4d*-amy | This study |
| p*yl.EXP1*-amy | *yl.EXP1* promoter inserted into *Spe*I/*Nde*I in p*yl.hp4d*-amy | This study |
| p*km.PDC1*-amy | Amylase gene inserted into *Kpn*I in pKm.PDC1-kmFBA1 | This study |
| p*km.FBA1*-amy | *km*.*FBA1* promoter inserted into *Sal*I/*Nde*I in p*km.PDC1*-amy | This study |
| p*km.TEF1*-amy | *km*.*TEF1* promoter inserted into *Sal*I/*Nde*I in p*km.PDC1*-amy | This study |
| p*km.TDH3*-amy | k*m*.*TDH3* promoter inserted into *Sal*I/*Nde*I in p*km.PDC1*-amy | This study |
| p*km.ENO1*-amy | k*m*.*ENO1* promoter inserted into *Sal*I/*Nde*I in p*km.PDC1*-amy | This study |
| p*yl.hp4d*-rfp | hp4d promoter for RFP | laboratory storage |
| p*yl.FBA1in*-rfp | RFP gene inserted into *Kpn*I in p*yl.FBA1in* | This study |
| p*yl.TEF1*-rfp | *yl*.*TEF1* promoter inserted into *Spe*I/*Nde*I in p*yl.FBA1in*-rfp | This study |
| p*yl.TDH1*-rfp | *yl*.*TDH1* promoter inserted into *Spe*I/*Nde*I in p*yl.FBA1in*-rfp | This study |
| p*yl.EXP1*-rfp | *yl*.*EXP1* promoter inserted into *Spe*I/*Nde*I in p*yl.FBA1in*-rfp | This study |
| p*km.PDC1*-rfp | RFP gene inserted into *Kpn*I in p*km.PDC1-km.FBA1* | This study |
| p*km.FBA1*-rfp | *km*.*FBA1* promoter inserted into *Sal*I/*Nde*I in p*km.PDC1*-rfp | This study |
| p*km.TEF1*-rfp | *km*.*TEF1* promoter inserted into *Sal*I/*Nde*I in p*km.PDC1*-rfp | This study |
| p*km.TDH3*-rfp | *km*.*TDH3* promoter inserted into *Sal*I/*Nde*I in p*km.PDC1*-rfp | This study |
| p*km.ENO1*-rfp | *km*.*ENO1* promoter inserted into *Sal*I/*Nde*I in p*km.PDC1*-rfp | This study |
| pET28a-rfp | RFP gene inserted into *Nde*I/*Xho*I in pET28a | This study |
| pET28a-amy | Amylase gene inserted into *Nde*I/*Xho*I in pET28a | This study |
| pXMJ19-*yl.TEF1*-rfp | Linear *yl*.*TEF1*-rfp inserted into *Apa*I/*Hin*dIII in pXMJ19 | This study |
| pXMJ19-*km.TEF1*-rfp | Linear *km*.*TEF1*-rfp inserted into *Apa*I/*Hin*dIII in pXMJ19 | This study |
| pXMJ19-*yl.TEF1*-amy | Linear *yl*.*TEF1*-amy inserted into *Apa*I/*Hin*dIII in pXMJ19 | This study |
| pXMJ19-*km.TEF1*-amy | Linear *km*.*TEF1*-amy inserted into *Apa*I/*Hin*dIII in pXMJ19 | This study |

**Table S2** Primers used in this study.

| Primers | Sequence (5’→3’), homologous arms are underlined and restriction sites are indicated in bold font |
| --- | --- |
| Broad-host vector-F | CAGTGTCTTGCGTCTCTTTATGC |
| Broad-host vector-R | TAACCGTATTACCGCCTTTGAGTG |
| p*yl.FBA1in*-F | ACCCGCAAGCTTGTCGAC**ACTAGT**GTAGCAACAACAGTGTACGCAG |
| p*yl.FBA1in*-R | AATTGCCTGCAGGGTACC**CATATG**GAAGAGCTGGGTTAGTTTGTGTAG |
| p*km.PDC1*-F | ACCCGCAAGCTTGTCGAC**ACTAGT**TCCAGCGAATATACAGCGTG |
| p*km.PDC1*-R | AATTGCCTGCAGGGTACC**CATATG**GCAATTATTTGGTTTGGGTG |
| p*km.PDC1*-*km.FBA1*-F | TGCTTCTCTTTGTGTGTAGTGTACGAGCGACAAACACACTCC |
| p*km.PDC1*-*km.FBA1*-R | GAGTTCAGGCTTTTTCAT**GAGCTC**TTAGAATTTATTGGTTATTTGTGTGCTAAAAG |
| p*yl.hp4d*-amy-F | ACCCGAAACTAAGCATATG**GGTACC**ATGCAGGTGCTGAACACTATG |
| p*yl.hp4d*-amy-R | TATCTGTTAATTGCCTGCAG**GGTACC**TCAGATTTTCTCCCAGATTGCGTAG |
| p*yl.FBA1in*-amy-F | identical to p*yl.FBA1in*-F |
| p*yl.FBA1in*-amy-R | TTCAGCACCTGCATGGTACC**CATATG**GAAGAGCTGGGTTAGTTTGTGTAG |
| p*yl.TEF1*-amy-F | ACCCGCAAGCTTGTCGAC**ACTAGT**TGGAAGTCGACCAGAGACCG |
| p*yl.TEF1*-amy-R | TTCAGCACCTGCATGGTACC**CATATG**TTTGAATGATTCTTATACTCAGAAGGAAATGC |
| p*yl.TDH1*-amy-F | ACCCGCAAGCTTGTCGAC**ACTAGT**CTCGGTAGTCGGAAAGAGC |
| p*yl.TDH1*-amy-R | TTCAGCACCTGCATGGTACC**CATATG**TGTTGATGTGTGTTTAATTCAAGAATG |
| p*yl.EXP1*-amy-F | ACCCGCAAGCTTGTCGAC**ACTAGT**ATATAAGGAGTTTGGCGCCCG |
| p*yl.EXP1*-amy-R | TTCAGCACCTGCATGGTACC**CATATG**TGCTGTAGATATGTCTTGTGTGTAAGG |
| p*km.PDC1*-amy-F | AACCAAATAATTGCCATATG**GGTACC**ATGCAGGTGCTGAACACTATG |
| p*km.PDC1*-amy-R | identical to p*yl.hp4d*-amy-R |
| p*km.FBA1*-amy-F | GCAGCCGGACCCGCAAGCTT**GTCGAC**GAGCGACAAACACACTCC |
| p*km.FBA1*-amy-R | TTCAGCACCTGCATGGTACC**CATATG**TTAGAATTTATTGGTTATTTGTGTGCTAAAAG |
| p*km.TEF1*-amy-F | GCAGCCGGACCCGCAAGCTT**GTCGAC**CAACGCATATGCTGCAATC |
| p*km.TEF1*-amy-R | TTCAGCACCTGCATGGTACC**CATATG**TTTAATGTTACTTCTCTTGGAGTTAG |
| p*km.TDH3*-amy-F | GCAGCCGGACCCGCAAGCTT**GTCGAC**GAAACTTGGGCCATGACAAAG |
| p*km.TDH3*-amy-R | TTCAGCACCTGCATGGTACC**CATATG**GTGATGTGTAAAAGTGTGTGTGTAC |
| p*km.ENO1*-amy-F | GCAGCCGGACCCGCAAGCTT**GTCGAC**AAGCCTTGAGCTTCGTGTTTATTCAC |
| p*km.ENO1*-amy-R | TTCAGCACCTGCATGGTACC**CATATG**GTAGTTTGTGTTTGTTGTTGTTGATGTG |
| p*yl.FBA1in*-rfp-F | CTAACCCAGCTCTTCCATATG**GGTACC**ATGGTGTCTAAGGGCGAAGAG |
| p*yl.FBA1in*-rfp-R | TATCTGTTAATTGCCTGCAG**GGTACC**TTACTTGTAGAGTTCGTCCATGCC |
| p*yl.TEF1*-rfp-F | identical to p*yl.TEF1*-amy-F |
| p*yl.TEF1*-rfp-R | GCCCTTAGACACCATGGTACC**CATATG**TTTGAATGATTCTTATACTCAGAAGGAAATGC |
| p*yl.TDH1*-rfp-F | identical to p*yl.TDH1*-amy-F |
| p*yl.TDH1*-rfp-R | GCCCTTAGACACCATGGTACC**CATATG**TGTTGATGTGTGTTTAATTCAAGAATG |
| p*yl.EXP1*-rfp-F | identical to p*yl.EXP1*-amy-F |
| p*yl.EXP1*-rfp-R | GCCCTTAGACACCATGGTACC**CATATG**TGCTGTAGATATGTCTTGTGTGTAAGG |
| p*km.PDC1*-rfp-F | AACCAAATAATTGCCATATG**GGTACC**ATGGTGTCTAAGGGCGAAGAG |
| p*km.PDC1*-rfp-R | identical to p*yl.FBA1in*-rfp-R |
| p*km.FBA1*-rfp-F | identical to p*km.FBA1*-amy-F |
| p*km.FBA1*-rfp-R | GCCCTTAGACACCATGGTACC**CATATG**TTAGAATTTATTGGTTATTTGTGTGCTAAAAG |
| p*km.TEF1*-rfp-F | identical to p*km.TEF1*-amy-F |
| p*km.TEF1*-rfp-R | GCCCTTAGACACCATGGTACC**CATATG**TTTAATGTTACTTCTCTTGGAGTTAG |
| p*km.TDH3*-rfp-F | identical to p*km.TDH3*-amy-F |
| p*km.TDH3*-rfp-R | GCCCTTAGACACCATGGTACC**CATATG**GTGATGTGTAAAAGTGTGTGTGTAC |
| p*km.ENO1*-rfp-F | identical to p*km.ENO1*-amy-F |
| p*km.ENO1*-rfp-R | GCCCTTAGACACCATGGTACC**CATATG**GTAGTTTGTGTTTGTTGTTGTTGATGTG |
| pET28a-rfp-F | CCTGGTGCCGCGCGGCAGC**CATATG**ATGGTGTCTAAGGGCGAAGAG |
| pET28a-rfp-R | GTGGTGGTGGTGGTGGTG**CTCGAG**TTACTTGTAGAGTTCGTCCATGCC |
| pET28a-amy-F | CCTGGTGCCGCGCGGCAGC**CATATG**ATGCAGGTGCTGAACACTATG |
| pET28a-amy-R | GTGGTGGTGGTGGTGGTG**CTCGAG**TCAGATTTTCTCCCAGATTGCGTAG |
| pXMJ19- *yl.TEF1*-rfp-F | GCGCCGAGACAGAACTTAAT**GGGCCC**TGGAAGTCGACCAGAGACCG |
| pXMJ19- *yl.TEF1*-rfp-R | GAGTCGACCTGCAGGCATGC**AAGCTT**TTACTTGTAGAGTTCGTCCATGCC |
| pXMJ19- *km.TEF1*-rfp-F | GCGCCGAGACAGAACTTAAT**GGGCCC**CAACGCATATGCTGCAATC |
| pXMJ19- *km.TEF1*-rfp-R | GAGTCGACCTGCAGGCATGC**AAGCTT**TTACTTGTAGAGTTCGTCCATGCC |
| pXMJ19- *yl.TEF1*-amy-F | GCGCCGAGACAGAACTTAAT**GGGCCC**TGGAAGTCGACCAGAGACCG |
| pXMJ19- *yl.TEF1*-amy-R | GAGTCGACCTGCAGGCATGC**AAGCTT**CAGATTTTCTCCCAGATTGCGTAG |
| pXMJ19- *km.TEF1*-amy-F | GCGCCGAGACAGAACTTAAT**GGGCCC**CAACGCATATGCTGCAATC |
| pXMJ19- *km.TEF1*-amy-R | GAGTCGACCTGCAGGCATGC**AAGCTT**CAGATTTTCTCCCAGATTGCGTAG |
| p*yl.FBA1in*-ck-F | GTAGCAACAACAGTGTACGCAG |
| p*yl.FBA1in*-ck-R | AGAATTATCACCGGCAAACTATCTG |
| p*km.PDC1*-ck-F | GCGTGAATAATGGAATGGCCTTG |
| p*km.PDC1*-ck-R | identical to p*yl.FBA1in*-ck-R |
| p*km.PDC1*-*km.FBA1*-ck-F | ATTTACGATGCCAATGCGAATAG |
| p*km.PDC1*-*km.FBA1*-ck-R | CAGAAGGCGAAATATCACAACAC |
| p*yl.hp4d*-amy-ck-F | TTGTCCCTTTCGGTCCTCATC |
| p*yl.hp4d*-amy-ck-R | GTATTCGAACACGGGCATCTCAC |
| p*yl.FBA1in*-amy-ck-F | identical to p*yl.FBA1in*-ck-F |
| p*yl.FBA1in*-amy-ck-R | CTTGCCCATCAGGAAGTTGTACC |
| p*yl.TEF1*-amy-ck-F | TGGAAGTCGACCAGAGACCG |
| p*yl.TEF1*-amy-ck-R | identical to p*yl.FBA1in*-amy-ck-R |
| p*yl.TDH1*-amy-ck-F | CATAAACCGGACGCAGTAGGATG |
| p*yl.TDH1*-amy-ck-R | identical to p*yl.FBA1in*-amy-ck-R |
| p*yl.EXP1*-amy-ck-F | CATGCTGTTCATCGTGGTTAATGC |
| p*yl.EXP1*-amy-ck-R | identical to p*yl.FBA1in*-amy-ck-R |
| p*km.PDC1*-amy-ck-F | identical to p*yl.hp4d*-amy-ck-F |
| p*km.PDC1*-amy-ck-R | identical to p*yl.hp4d-*amy-ck-R |
| p*km.FBA1*-amy-ck-F | CTGGTTTTTGTTTTTGTCTCTCCCC |
| p*km.FBA1*-amy-ck-R | identical to p*yl.FBA1in*-amy-ck-R |
| p*km.TEF1*-amy-ck-F | CCAGAAACAACCGCACAAATACG |
| p*km.TEF1*-amy-ck-R | identical to p*yl.FBA1in*-amy-ck-R |
| p*km.TDH3*-amy-ck-F | GAAACTTGGGCCATGACAAAG |
| p*km.TDH3*-amy-ck-R | identical to p*yl.FBA1in*-amy-ck-R |
| p*km.ENO1*-amy-ck-F | AGCTTCGTGTTTATTCACTCTGTACG |
| p*km.ENO1*-amy-ck-R | identical to p*yl.FBA1in*-amy-ck-R |
| p*yl.FBA1in*-rfp-ck-F | CATGGTGTCTAAGGGCGAAGAG |
| p*yl.FBA1in*-rfp-ck-R | identical to p*yl.hp4d*-amy-ck-R |
| p*yl.TEF1*-rfp-ck-F | identical to p*yl.TEF1*-amy-ck-F |
| p*yl.TEF1*-rfp-ck-R | CCATCAGCGGGATACATCATCTC |
| p*yl.TDH1*-rfp-ck-F | identical to p*yl.TDH1*-amy-ck-F |
| p*yl.TDH1*-rfp-ck-R | identical to p*yl.TEF1*-rfp-ck-R |
| p*yl.EXP1*-rfp-ck-F | identical to p*yl.EXP1*-amy-ck-F |
| p*yl.EXP1*-rfp-ck-R | identical to p*yl.TEF1*-rfp-ck-R |
| p*km.PDC1*-rfp-ck-F | identical to p*yl.FBA1in*-rfp-ck-F |
| p*km.PDC1*-rfp-ck-R | identical to p*yl.hp4d*-amy-ck-R |
| p*km.FBA1*-rfp-ck-F | identical to p*km.FBA1*-amy-ck-F |
| p*km.FBA1*-rfp-ck-R | identical to p*yl.TEF1*-rfp-ck-R |
| p*km.TEF1*-rfp-ck-F | identical to p*km.TEF1*-amy-ck-F |
| p*km.TEF1*-rfp-ck-R | identical to p*yl.TEF1*-rfp-ck-R |
| p*km.TDH3*-rfp-ck-F | identical to p*km.TDH3*-amy-ck-F |
| p*km.TDH3*-rfp-ck-R | identical to p*yl.TEF1*-rfp-ck-R |
| p*km.ENO1*-rfp-ck-F | identical to p*km.ENO1*-amy-ck-F |
| p*km.ENO1*-rfp-ck-R | identical to p*yl.TEF1*-rfp-ck-R |
| pET28a-rfp-ck-F | CATGGTGTCTAAGGGCGAAGAG |
| pET28a-rfp-ck-R | CAACTCAGCTTCCTTTCGGGC |
| pET28a-amy-ck-F | TTGTCCCTTTCGGTCCTCATC |
| pET28a-amy-ck-R | identical to pET28a-rfp-ck-R |
| pXMJ19- *yl.TEF1*-rfp-ck-F | identical to p*yl.FBA1in*-rfp-ck-F |
| pXMJ19- *yl.TEF1*-rfp-ck-R | TGAAGCATTTATCAGGGTTATTGTCTC |
| pXMJ19- *km.TEF1*-rfp-ck-F | identical to p*yl.FBA1in*-rfp-ck-F |
| pXMJ19- *km.TEF1*-rfp-ck-R | identical to pXMJ19-*yl.TEF1*-rfp-ck-R |
| pXMJ19- *yl.TEF1*-amy-ck-F | identical to p*yl.hp4d*-amy-ck-F |
| pXMJ19- *yl.TEF1*-amy-ck-R | identical to pXMJ19-*yl.TEF1*-rfp-ck-R |
| pXMJ19- *km.TEF1*-amy-ck-F | identical to p*yl.hp4d*-amy-ck-F |
| pXMJ19- *km.TEF1*-amy-ck-R | identical to pXMJ19-*yl.TEF1*-rfp-ck-R |

**Table S3** The 540 nm absorbance of five-time diluted DNS reaction mixture of the controls for preliminary amylase activity assays.

| **540nm** [**absorbance**](javascript:;)**（A）** | **Replication 1** | **Replication 2** | **Replication 3** |
| --- | --- | --- | --- |
| Control (distilled water instead of crude amylase extract) | 0.1197 | 0.1131 | 0.1019 |
| Crude amylase extract of the strain YL-no.promoter-amy | 0.0974 | 0.1106 | 0.0978 |
| Crude amylase extract of the strain YL-*yl.hp4d*-no.gene | 0.1028 | 0.0944 | 0.0922 |
| Crude amylase extract of the wild type *Y. lipolytica* msn4 | 0.1211 | 0.1029 | 0.0999 |
| Crude amylase extract of the strain KM-no.promoter-amy | 0.0945 | 0.0940 | 0.1050 |
| Crude amylase extract of the strain KM-*km.PDC1*-no.gene | 0.0953 | 0.0985 | 0.0960 |
| Crude amylase extract of the wild type *K. marxianus* CGMCC2.1977 | 0.1021 | 0.1120 | 0.1100 |

^YL:^ *^Y. lipolytica^*^; KM:^ *^K. marxianus^*^;^ *^yl.hp4d^*^: hybrid promoter contains four UAS1 tandem elements based on the minimal LEU2 promoter (^*^UAS1B4-leum)^*^;^ *^km.PDC1^*^: the promoter of pyruvate decarboxylase.^

**Table S4** The fluorescence intensity of 1OD different control strains for preliminary RFP quantitative fluorescence experiments.

| **Fluorescence Intensity (RFU)** | **Replication 1** | **Replication 2** | **Replication 3** |
| --- | --- | --- | --- |
| The strain YL-no.promoter-rfp (Gain value: 80) | 377 | 372 | 359 |
| The strain YL-*yl.hp4d*-no.gene (Gain value: 80) | 364 | 370 | 365 |
| The wild type *Y. lipolytica* msn4 (Gain value: 80) | 372 | 373 | 382 |
| The strain KM-no.promoter-rfp (Gain value: 80) | 447 | 450 | 445 |
| The strain KM-km*.PDC1*-no.gene (Gain value: 80) | 437 | 447 | 443 |
| The wild type *K. marxianus* CGMCC2.1977 (Gain value: 80) | 454 | 464 | 465 |
| The strain DE3-no.promoter-rfp (Gain value:70) | 74 | 74 | 73 |
| The strain DE3-*km.TEF1*-no.gene (Gain value: 70) | 73 | 74 | 74 |
| The wild type *E. coli BL21*(DE3) (Gain value: 70) | 76 | 78 | 75 |
| The strain PP-no.promoter-rfp (Gain value: 80) | 237 | 230 | 223 |
| The strain PP-*yl.hp4d*-no.gene (Gain value: 80) | 229 | 230 | 230 |
| The wild type *Pichia pastoris* GS115 (Gain value: 80) | 295 | 248 | 251 |
| The strain CG-pXMJ19-no.promoter-rfp (Gain value: 80) | 352 | 477 | 352 |
| The strain CG-pXMJ19-*km.TEF1*-no.gene (Gain value: 80) | 347 | 362 | 370 |
| The wild type *Corynebacterium glutamicum* ATCC13032 (Gain value: 80) | 451 | 451 | 445 |

^YL:^ *^Y. lipolytica^*^; KM:^ *^K. marxianus^*^; DE3:^ *^E. coli^*^; PP:^ *^Pichia pastoris^*^; CG:^ *^C. glutamicum^*^;^ *^yl.hp4d^*^: hybrid promoter contains four UAS1 tandem elements based on the minimal LEU2 promoter (^*^UAS1B4-leum)^*^;^ *^km.PDC1^*^: the promoter of pyruvate decarboxylase;^ *^km.TEF1^*^: the promoter of translation elongation factor EF alpha-1.^


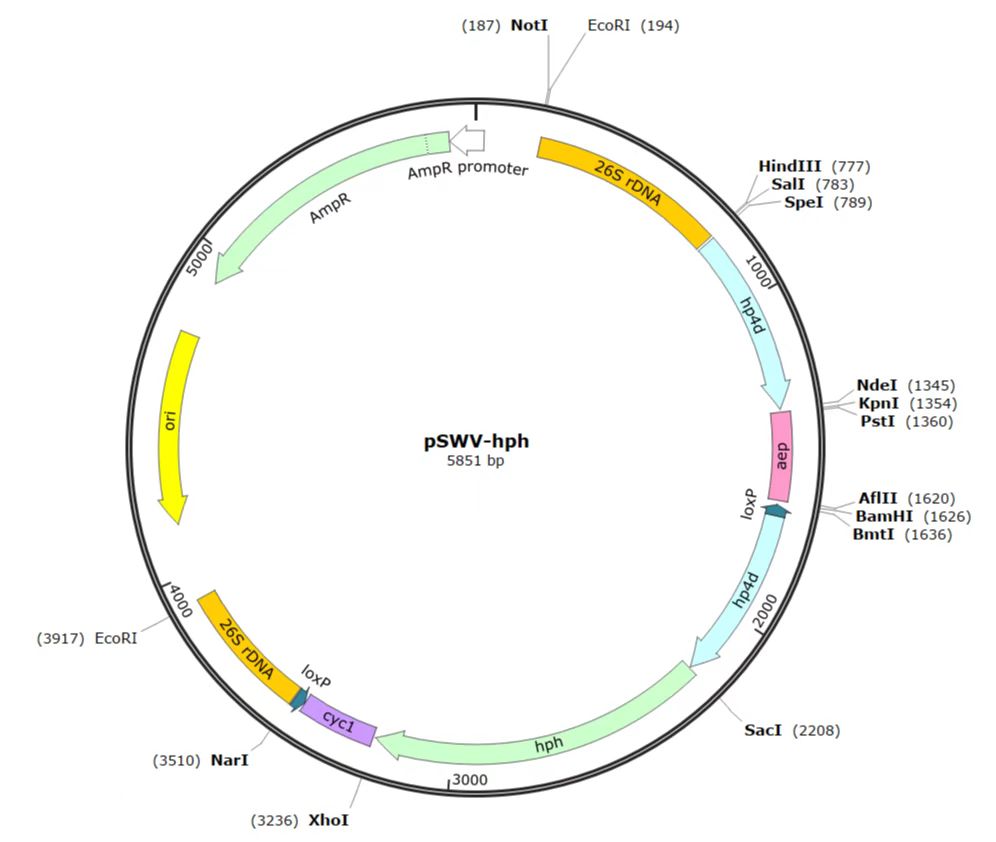


**Fig. S1**

**Fig. S2**

**Fig. S3**

**Sequences of five *Y. lipolytica* constitutive promoters and five *K. marxianus* constitutive promoters**

*yl*.*hp4d*

CTGAGGTGTCTCACAAGTGCCGTGCAGTCCCGCCCCCACTTGCTTCTCTTTGTGTGTAGTGTACGTACATTATCGAGACCGTTGTTCCCGCCCACCTCGATCCGGCTGAGGTGTCTCACAAGTGCCGTGCAGTCCCGCCCCCACTTGCTTCTCTTTGTGTGTAGTGTACGTACATTATCGAGACCGTTGTTCCCGCCCACCTCGATCCGGCTGAGGTGTCTCACAAGTGCCGTGCAGTCCCGCCCCCACTTGCTTCTCTTTGTGTGTAGTGTACGTACATTATCGAGACCGTTGTTCCCGCCCACCTCGATCCGGCTGAGGTGTCTCACAAGTGCCGTGCAGTCCCGCCCCCACTTGCTTCTCTTTGTGTGTAGTGTACGTACATTATCGAGACCGTTGTTCCCGCCCACCTCGATCCGGCACGGGCAAAAGTGCGTATATATACAAGAGCGTTTGCCAGCCACAGATTTTCACTCCACACACCACATCACACATACAACCACACACATCCACAATGGAACCCGAAACTAAG

*yl*.*FBA1in*

GTAGCAACAACAGTGTACGCAGTACTATAGAGGAACAATTGCCCCGGAGAAGACGGCCAGGCCGCCTAGATGACAAATTCAACAACTCACAGCTGACTTTCTGCCATTGCCACTAGGGGGGGGCCTTTTTATATGGCCAAGCCAAGCTCTCCACGTCGGTTGGGCTGCACCCAACAATAAATGGGTAGGGTTGCACCAACAAAGGGATGGGATGGGGGGTAGAAGATACGAGGATAACGGGGCTCAATGGCACAAATAAGAACGAATACTGCCATTAAGACTCGTGATCCAGCGACTGACACCATTGCATCATCTAAGGGCCTCAAAACTACCTCGGAACTGCTGCGCTGATCTGGACACCACAGAGGTTCCGAGCACTTTAGGTTGCACCAAATGTCCCACCAGGTGCAGGCAGAAAACGCTGGAACAGCGTGTACAGTTTGTCTTAGCAAAAAGTGAAGGCGCTGAGGTCGAGCAGGGTGGTGTGACTTGTTATAGCCTTTAGAGCTGCGAAAGCGCGTATGGATTTGGCTCATCAGGCCAGATTGAGGGTCTGTGGACACATGTCATGTTAGTGTACTTCAATCGCCCCCTGGATATAGCCCCGACAATAGGCCGTGGCCTCATTTTTTTGCCTTCCGCACATTTCCATTGCTCGGTACCCACACCTTGCTTCTCCTGCACTTGCCAACCTTAATACTGGTTTACATTGACCAACATCTTACAAGCGGGGGGCTTGTCTAGGGTATATATAAACAGTGGCTCTCCCAATCGGTTGCCAGTCTCTTTTTTCCTTTCTTTCCCCACAGATTCGAAATCTAAACTACACATCACACAATGCCTGTTACTGACGTCCTTAAGCGAAAGTCCGGTGTCATCGTCGGCGACGATGTCCGAGCCGTGAGTATCCACGACAAGATCAGTGTCGAGACGACGCGTTTTGTGTAATGACACAATCCGAAAGTCGCTAGCAACACACACTCTCTACACAAACTAACCCAGCTCTTC

*yl*.*TEF1*

TGGAAGTCGACCAGAGACCGGGTTGGCGGCGTATTTGTGTCCCAAAAAACAGCCCCAATTGCCCCAATTGACCCCAAATTGACCCAGTAGCGGGCCCAACCCCGGCGAGAGCCCCCTTCACCCCACATATCAAACCTCCCCCGGTTCCCACACTTGCCGTTAAGGGCGTAGGGTACTGCAGTCTGGAATCTACGCTTGTTCAGACTTTGTACTAGTTTCTTTGTCTGGCCATCCGGGTAACCCATGCCGGACGCAAAATAGACTACTGAAAATTTTTTTGCTTTGTGGTTGGGACTTTAGCCAAGGGTATAAAAGACCACCGTCCCCGAATTACCTTTCCTCTTCTTTTCTCTCTCTCCTTGTCAACTCACACCCGAAATCGTTAAGCATTTCCTTCTGAGTATAAGAATCATTCAAA

*yl*.*TDH1*

CTCGGTAGTCGGAAAGAGCCGGGACCGGCCGGCGAGCATAAACCGGACGCAGTAGGATGTCCTGCACGGGTCTTTTTGTGGGGTGTGGAGAAAGGGGTGCTTGGAGATGGAAGCCGGTAGAACCGGGCTGCTTGGGGGGATTTGGGGCCGCTGGGCTCCAAAGAGGGGTAGGCATTTCGTTGGGGTTACGTAATTGCGGCATTTGGGTCCTGCGCGCATGTCCCATTGGTCAGAATTAGTCCGGATAGGAGACTTATCAGCCAATCACAGCGCCGGATCCACCTGTAGGTTGGGTTGGGTGGGAGCACCCCTCCACAGAGTAGAGTCAAACAGCAGCAGCAACATGATAGTTGGGGGTGTGCGTGTTAAAGGAAAAAAAAAGAAGCTTGGGTTATATTCCCGCTCTATTTAGAGGTTGCGGGATAGACGCCGACGGAGGGCAATGGCGCCATGGAACCTTGCGGATATCGATACGCCGCGGCGGACTGCGTCCGAACCAGCTCCAGCAGCGTTTTTTCCGGGCCATTGAGCCGACTGCGACCCCGCCAACGTGTCTTGGCCCACGCACTCATGTCATGTTGGTGTTGGGAGGCCACTTTTTAAGTAGCACAAGGCACCTAGCTCGCAGCAAGGTGTCCGAACCAAAGAAGCGGCTGCAGTGGTGCAAACGGGGCGGAAACGGCGGGAAAAAGCCACGGGGGCACGAATTGAGGCACGCCCTCGAATTTGAGACGAGTCACGGCCCCATTCGCCCGCGCAATGGCTCGCCAACGCCCGGTCTTTTGCACCACATCAGGTTACCCCAAGCCAAACCTTTGTGTTAAAAAGCTTAACATATTATACCGAACGTAGGTTTGGGCGGGCTTGCTCCGTCTGTCCAAGGCAACATTTATATAAGGGTCTGCATCGCCGGCTCAATTGAATCTTTTTTCTTCTTCTCTTCTCTATATTCATTCTTGAATTAAACACACATCAACA

*yl*.*EXP1*

ATATAAGGAGTTTGGCGCCCGTTTTTTCGAGCCCCACACGTTTCGGTGAGTATGAGCGGCGGCAGATTCGAGCGTTTCCGGTTTCCGCGGCTGGACGAGAGCCCATGATGGGGGCTCCCACCACCAGCAATCAGGGCCCTGATTACACACCCACCTGTAATGTCATGCTGTTCATCGTGGTTAATGCTGCTGTGTGCTGTGTGTGTGTGTTGTTTGGCGCTCATTGTTGCGTTATGCAGCGTACACCACAATATTGGAAGCTTATTAGCCTTTCTATTTTTTCGTTTGCAAGGCTTAACAACATTGCTGTGGAGAGGGATGGGGATATGGAGGCCGCTGGAGGGAGTCGGAGAGGCGTTTTGGAGCGGCTTGGCCTGGCGCCCAGCTCGCGAAACGCACCTAGGACCCTTTGGCACGCCGAAATGTGCCACTTTTCAGTCTAGTAACGCCTTACCTACGTCATTCCATGCATGCATGTTTGCGCCTTTTTTCCCTTGCCCTTGATCGCCACACAGTACAGTGCACTGTACAGTGGAGGTTTTGGGGGGGTCTTAGATGGGAGCTAAAAGCGGCCTAGCGGTACACTAGTGGGATTGTATGGAGTGGCATGGAGCCTAGGTGGAGCCTGACAGGACGCACGACCGGCTAGCCCGTGACAGACGATGGGTGGCTCCTGTTGTCCACCGCGTACAAATGTTTGGGCCAAAGTCTTGTCAGCCTTGCTTGCGAACCTAATTCCCAATTTTGTCACTTCGCACCCCCATTGATCGAGCCCTAACCCCTGCCCATCAGGCAATCCAATTAAGCTCGCATTGTCTGCCTTGTTTAGTTTGGCTCCTGCCCGTTTCGGCGTCCACTTGCACAAACACAAACAAGCATTATATATAAGGCTCGTCTCTCCCTCCCAACCACACTCACTTTTTTGCCCGTCTTCCCTTGCTAACACAAAAGTCAAGAACACAAACAACCACCCCAACCCCCTTACACACAAGACATATCTACAGCA

*km*.*PDC1* TCCAGCGAATATACAGCGTGAATAATGGAATGGCCTTGTATTCGTTTTTTCCGAGAGAAAAAAACGGGCTTCGGTGAAAATCGGGTGAATATGCAACTAGCGGGACGAATGCTCTGGAAATGCATATCCTATGCAACTAGCGGGATGAACAAATCTCACCCCAGAATTCGCAGGAAAAAACAGGAAAAAAAAAAAGAAGGCCACCACGGCCACAAAGACCACAAAGACCACAAAAAAAAACAAAAAACAACCGTCCCAGCTTCCAGTGTTTGGAATACTGGAACACAGGAAGCCGCATAAGAGTGGGCGTTGCACAGGAAGCCAGGCCCAGAAGCCCCAGAGTTACTTTTTTTTTTTTGTTTTTTCCTTCTGTTCGCTGTGCCCGCATCAGATGATGCGCCTTTATTTACGATGCCAATGCGAATAGCACCAGTGAGAGCACCAGTAAAAGCATACGCATACACATACACACATAGAGCAAGCAAGCAGGCTAGCAACCAGGAAAGGCTGCCAGTGACTGCTACTGGGTGTCTAAGAACCGTAGGGCGGATTATTGTTGCGGTGGTTGGTTGCGGGTGGTTATGCGATGGTACGGTGCAGAATCGTACGGTGTTGGTTATGGAATTAGTATGGGTATGTGATATGTGGTAATATGTGATATTGGGTTATTGTGATTTGGAATACTGAATATCGAATATGGGATATGGAATATGGCCATGGCATGGTATGGTATGGGATGGGAGTATTCTATTTTATTTTATTTTATTCTGGTTCCTGCGTTTAGGGTAGGGTAGGAAGAAGGTGAGTGCTTTTGTATATAAGTGGAGTGTCTGGATCAGTTTTGTGGATTGTGAATGTTAGTTTCCCCTTTAATGTATATTTGTATTATTTGCTTTTGAGTACTCAATAACCAAGCACAACTACTAGTTTTAAAGGATCCATCCTCTTAAACAGTACAAATCGCAAAGAAAAGCTCCACACCCAAACCAAATAATTGC

*km*.*FBA1*

GAGCGACAAACACACTCCCACATACATCTTTCCCGGAGGCAGAAACAAAATAAGAACAACAACAACACACCAACACGCCAACCATAGTAACCCACACGCTTAAACAACCAGTATCAGACCGGTACCCAACTTCTACGGCCGGCGGCGGCTGGCAGCGGCCTCGAGCATCCTCTATTCCCTCACCTCTTCTCTCGCTGTTGTACAACTTACAGGAATTCCCCTCTCTCCTCGAACTGGAAGCCGTGTTTCACGTGAGTGGTGGTACGATACCCGGTGGGAACCCTGTGTTGTGATATTTCGCCTTCTGGTTTTTGTTTTTGTCTCTCCCCTCTGGCTTCCAATACCGGAAAATTTGGAAATTTTGGAAAAACAGGAAATCACAAAAAAACAGGAGAACCTGGAAAACGTAACGAAACCAAATAGTGGAACCCAACTGAATCCGCCGTAGACCAGATCAACCCACATGATTAAACGTCGCCCTCGACTTCCAGTGGTACTTCCAGTACCAATTCCAGTACCACTTTGAACAACTCTCCATAACACTTCCAATTCCACTTACAGTGTATGTGTGTGTATGTGTGTGCGTGTGTGGACAGGTTTCTTGCGCTTCTTGTACTTCTGGTGATGATAGATGGTGGATTCTCGGTACAGGAATTGGCTCAGGCTTTTTTTTTTTCACATCGATTTCAGTTTGGGCTTTCCCTATGCATTCATTATATTGTGATTGCATTGAGCTTTGGAAATTTTTCATACTCTTCGAAATCATATATAATAAGTTACAATGTGAGTTATTCTCTCCTTCCTGGTTGTTGGTGTAAGCATCATTTAACTAGTCCCTTTTTTTTAAAGTTATTTAATATATATTTTTATTTTGTCCCAAACACCAAGAAGTAATCTTTACTTTTAGCACACAAATAACCAATAAATTCTAA

*km*.*TEF1*

CAACGCATATGCTGCAATCACCCAACGGAATTAATTAATTAAGTAGTTACTTACAGTATTGTAATGCCATCCCAGCGTATCCCAGCCTAGTGTATCCCAGCCTAGCCTAGCCTAGGCCAAACCTAGGCCAAACCTAGGCCAAACCTAGCCCTCTCTAGCCTAGCGCCCAGCAGAAACACCGATGAAGCAAAGAAGTAACAGCAGGAAAGAAAAACAAACACAACAAAAAAAAACAAGCAGCATAGCATCAACAGAAATTTCTAAAGAGAACCAAATTCACCCCAGAAACAACCGCACAAATACGACATCCATCCACCTTTCTTTTATCTTCTTTTTCTGATCTGATAATTAGTTTCATATACAATACGTAGAAACAGGCGCACAGCACCCAGACCTGGCTTCTGCCCCAGTGTATAAGCAATGTAGCATAATTGGAAAAAAAAACGAAAAATACCGAAAATAAGTGGGAAGCTGGGCCACAGGAGTGGGGCGGGATGCGACTGGTTCTGAGCGGGACCGGGTAATAAGGTTGAAAAACTTTGAATTGATGGAATAAGTAACTTCTTTCTTTTCGCTGGCGGGAGAAGGAAAAAAAAAAATTTTTTTTCCTTCTGTTTAGTACTGGGACATTGAGAAGGCGTGTCAATTTTGAATAATTAGAGTGGTCAAAAAAATTTTTTTTGCTTGGGATACCCTTTTTCGATAATGTAAATTTTTTTTGAATATAAAAGGAGATTGAAAAATTTTTTCTAGCAGAAATGTTTTCAAGTTTTAATTGCAAGTTTCGTTTGAGTATTCAGTTGTATTTTAGTTGATTTGTAGTTTATTTACTAGTATTCTCATAGTTCTAACTCCAAGAGAAGTAACATTAAA

*km*.*TDH3*

GAAACTTGGGCCATGACAAAGGCCATGGAAAATGGTAGTACCATGGTAGTACCATGGTACTACTAGTGGTGGTGGCATTAGTGGTACCAGAACCACCTGTTGATTGATGCTGGGACTCCTGTATTTTGGTTAGGGCCTGTCTGTTGGGGCCTGTCAAACAACCAACAACACTCTTCCATATTCTGTTCTATCCAGCTAGCTAGCTAGCTAGCTGTCTTTCTCTCTCGTATGACATGGCCTAAGGCCATGTCATGTCACACATACACACTGTTTCCCACTGCTTCCCACTGTTTCCACTGGTTTACCGTTTCAGTAAATATCCAACATGCGATCATGCCAATATTCTGCCAATATTCTGCCAAATGTTCTTTCTGCCATTCTGCCATATATAAAGACCACATTTGATATCCAATTTCCCAATTCCAAATGTATTAGAATAGAATAGTCTATATTATACTCTATACAATTTTATAGTCCCTCCCAAACCAAGTCTTTTAGATTTAACAACAGTACACACACACTTTTACACATCAC

*km*.*ENO1*

AAGCCTTGAGCTTCGTGTTTATTCACTCTGTACGGGCGGTTCTAGCGTGTCTGGGAATCCAAAGAGGAAGGGCCGTGTTTCGTGCAGGCATTTTAGTGGATGCCCGGGATGTAAGCTATTCGGTTACAGAAAATCAGGTTGTGATTCCAGTATGAGGAATAGTGGATGCCAGGTTGTGCGAGCATCACCAATATCATGGGAAATGCGAAATGGGCATGAGCCCAACCAACACAACACAATACCATACGCCAAATGGTAGCCTGAAAAAAAAAAAAAAGGTCTTGCCAGACCACTAAACTCGAGGTAGAACAAGACTGAGAAAGAGTGTGTGATCCCTTTGGTGGTAGTAATTTTTTTTTTTTTTTTTTTCAAGTTTCCAGCATCCCAAACCGAAACCCAACATCATCATGATCCCATCCCATGCCTATGCCCATGCCACATTCATTCATTGCACAACACACAACGTAGTGGACGACAGCATAGCATCAGTTAACTAACGGCCACTTGTTTTTTTTTCTTCCATTTTTTCGTTTTGTTCTAGCAACAATGAGTTCTAATTTTTTTGTTATAAAAGGGACAGTTAAGGTATATGTAAGGTTTCTTGTTCCATTTGGGTTAGAGTTTTGTAGTATTAGTTTTGGTTCTTAGTCCTTTTGGCCTAAGAAACCAGTAAGAATTCTTCTGATTTTCTTTACAAACACATCAACAACAACAAACACAAACTAC

**Sequences of reporter genes α-amylase and RFP**

α-amylase gene

ATGCAGGTGCTGAACACTATGGTGAACAAACACTTCTTGTCCCTTTCGGTCCTCATCGTCCTCCTTGGCCTCTCCTCCAACTTGACAGCCGGGCAAGTCCTGTTTCAGGGATTCAACTGGGAGTCGTGGAAGGAGAATGGCGGGTGGTACAACTTCCTGATGGGCAAGGTGGACGACATCGCCGCAGCCGGCATCACCCACGTCTGGCTCCCTCCGCCGTCTCACTCTGTCGGCGAGCAAGGCTACATGCCTGGGCGGCTGTACGATCTGGACGCGTCTAAGTACGGCAACGAGGCGCAGCTCAAGTCGCTGATCGAGGCGTTTCATGGCAAGGGCGTCCAGGTGATCGCCGACATCGTCATCAACCACCGCACGGCGGAGCACAAGGACGGACGCGGCATCTACTGCCTCTTCGAGGGCGGGACGCCCGACTCCCGCCTCGACTGGGGACCGCACATGATCTGCCGCGACGACCCCTACGGCGATGGCACCGGCAACCCGGACACCGGCGCCGACTTCGCCGCCGCGCCGGACATCGACCACCTCAACAAGCGCGTCCAGCGGGAGCTGATTGGCTGGCTCGACTGGCTCAAGATGGACATCGGCTTCGACGCGTGGCGCCTCGACTTCGCCAAGGGCTACTCCGCCGACATGGCAAAGATCTACATCGACGCCACCGAGCCGAGCTTCGCCGTGGCCGAGATATGGACGTCTATGGCGAACGGCGGGGACGGCAAGCCGAACTACGACCAGAACGCGCACCGGCAGGAGCTGGTCAACTGGGTCGATCGTGTCGGCGGCGCCAACAGCAACGGCACGGCGTTCGACTTCACCACCAAGGGCATCCTCAACGTCGCCGTGGAGGGCGAGCTGTGGCGCCTGCGCGGCGAGGACGGCAAGGCGCCCGGCATGATCGGGTGGTGGCCGGCCAAGGCGACGACCTTCGTGGACAACCACGACACCGGCTCGACGCAGCACCTGTGGCCGTTCCCCTCCGACAAGGTCATGCAGGGCTACGCATACATCCTCACCCACCCCGGCAACCCATGCATCTTCTACGACCATTTCTTCGATTGGGGTCTCAAGGAGGAGATCGAGCGCCTGGTGTCAATCAGAAACCGGCAGGGGATTCACCCGGCGAGCGAGCTGCGCATCATGGAAGCTGACAGCGATCTCTACCTCGCGGAGATTGATGGCAAGGTGATCACAAAGATTGGACCAAGATACGACGTCGAACACCTCATCCCCGAAGGCTTCCAGGTCGTCGCGCACGGTGATGGCTACGCAATCTGGGAGAAAATCTGA

RFP gene

ATGGTGTCTAAGGGCGAAGAGCTGATTAAGGAAAATATGCGAATGAAGGTGGTCATGGAAGGTTCCGTCAACGGCCACCAATTCAAATGCACTGGTGAAGGAGAAGGCAATCCTTACATGGGAACTCAAACCATGCGAATCAAAGTCATCGAGGGAGGACCCCTGCCCTTTGCCTTTGACATTCTGGCTACCTCCTTCATGTATGGCTCCCGAACTTTTATCAAGTACCCTAAAGGCATTCCTGATTTCTTCAAACAGTCCTTTCCTGAGGGTTTTACTTGGGAACGAGTTACCCGATACGAAGATGGTGGAGTCGTCACCGTCATGCAGGACACCTCCCTGGAGGATGGCTGTCTCGTTTACCACGTCCAAGTCCGAGGCGTGAACTTTCCCTCCAATGGTCCCGTGATGCAGAAGAAGACCAAGGGTTGGGAGCCTAATACTGAGATGATGTATCCCGCTGATGGTGGTCTGCGAGGATACACTCACATGGCTCTGAAAGTTGATGGTGGTGGACATCTGTCTTGCTCTTTCGTTACTACTTACCGATCTAAAAAGACCGTCGGCAACATCAAGATGCCCGGTATCCATGCCGTTGACCACCGACTGGAACGACTGGAGGAATCTGACAATGAAATGTTCGTTGTTCAACGAGAACACGCTGTTGCCAAGTTCGCTGGCCTGGGTGGTGGCATGGACGAACTCTACAAGTAA
